# Responses and assembly mechanisms of planktonic bacterial and fungal communities in the upper Chishui River during dry and wet seasons

**DOI:** 10.3389/fmicb.2026.1949002

**Published:** 2026-10-20

**Authors:** Defu Liu, Wei Zheng, Hao Wen, Chaohuan Yang, Die Qu, Kaimei Zhang, Yongjie Xie, Guiliang Ji, Zhenyue Feng

**Affiliations:** 1 College of Agronomy and Life Sciences, Zhaotong University, Zhaotong, China; 2 Yunnan Engineering Research Center of Green Planting and Processing of Gastrodia, Zhaotong University, Zhaotong, China; 3 Advanced Institute of Ecological Agriculture and Biodiversity on the Yunnan-Guizhou Plateau, Zhaotong University, Zhaotong, China; 4 Yunnan Key Laboratory of Smart Villages and Agri-Cultural-Tourism Integration, Zhaotong University, Zhaotong, China; 5 College of Agronomy and Biotechnology, Yunnan Agricultural University, Kunming, China

**Keywords:** anthropogenic disturbance, bioindicator, Chishui River, community assembly, karst stream, planktonic bacteria, planktonic fungi, seasonal dynamics

## Abstract

The upper Chishui River in Zhaotong, Yunnan, is a typical karst headwater stream and remains the only undammed first-order tributary of the upper Yangtze River. Agricultural and urban land use creates a distinct longitudinal nutrient gradient. We investigated the responses of planktonic bacteria and fungi to seasonal hydrology and anthropogenic disturbances, as well as their community assembly processes. Surface water samples were collected in the dry season (January 2025) and wet season (May 2025) from three sites: an upstream least-disturbed reference site (CSY), a midstream site affected by agricultural and township runoff (GZ), and a downstream site receiving municipal wastewater discharges (EL). Using high-throughput amplicon sequencing of bacterial 16S rRNA genes and fungal ITS2 regions, we found that total nitrogen and total phosphorus increased downstream; seasonal hydrological variation altered nutrient concentrations but did not shift the overall spatial pattern. Nutrient and carbonate-related variables (pH and TP) were correlated with the community structure of both bacteria and fungi. Alpha diversity was highest at the upstream site. At midstream and downstream sites, species richness increased while community evenness declined in the dry season. Microbial community composition exhibited clear seasonal turnover: *Flavobacterium* dominated upstream in the wet season, while *Limnohabitans* prevailed at the midstream site in the dry season. Upstream fungal communities were enriched in terrestrial saprotrophs and plant pathogens, whereas Rozellomycota predominated downstream. Functional predictions based on Phylogenetic Investigation of Communities by Reconstruction of Unobserved States 2 (PICRUSt2) and FUNGuild (Fungi + Functional + Guild) indicated that stress-response pathways were relatively abundant in the wet season. In contrast, carbohydrate and amino acid metabolism pathways were relatively more abundant in the dry season. Fungal trophic guild diversity at midstream and downstream sites also increased in the dry season. Infer Community Assembly Mechanisms by Phylogenetic-bin-based null model analysis (iCAMP) indicated that homogeneous selection predominantly structured bacterial communities across both seasons. By comparison, fungal communities shifted from homogeneous selection in the wet season to dominance by stochastic processes (e.g., ecological drift) in the dry season. Collectively, these findings suggest that bacteria and fungi respond differently to anthropogenic stress and seasonal hydrology in karst headwaters, with substantial seasonal divergence in their core community assembly mechanisms. Rozellomycota and certain fungal trophic guilds may serve as candidate bioindicators of chronic disturbance, pending further validation. These results provide a foundation for ecological health assessment and water resource conservation in karst headwater streams.

## 1 Introduction

The Chishui River (27°20′–28°50′ N, 104°45′–106°51′ E) is the only undammed first-order tributary of the upper Yangtze River. It rises in Chishuiyuan Township, Zhenxiong County, Yunnan Province, and flows through Yunnan, Guizhou, and Sichuan before joining the Yangtze at Hejiang, Sichuan (Wang et al., 2024). The basin supplies drinking water, sustains traditional liquor production, and harbors endemic fish species (Feng et al., 2016; Xiao et al., 2025). The upper Zhaotong reach examined here lies in the core of the Wumeng Mountain karst region, where widespread carbonate rocks create strong surface water–groundwater connectivity. Pollutants and microbes can be transported rapidly through karst conduits, making this system more sensitive to anthropogenic and hydrological perturbations than typical non-karst rivers. This sensitivity, in turn, makes it a valuable natural setting for studying microbial community assembly (Wu et al., 2022). Elevation in the reach ranges from 630 to 2,416 m, with headwater zones at 1,500–1,700 m. The climate is northern subtropical humid, with a mean annual temperature of 11.3 °C and precipitation of 900–915 mm. The catchment has high forest coverage and serves as the principal water conservation zone for the basin.

Bacteria are fundamental components of ecosystems and play a vital role in global biogeochemical cycling (Ruiz-González et al., 2015; Kuypers et al., 2018; Fan et al., 2021). Planktonic microorganisms drive biogeochemical cycles in aquatic ecosystems by mediating organic matter decomposition, pollutant transformation, and water quality maintenance (Fan et al., 2016; Chen et al., 2018; Pernthaler and Amann, 2005). Bacteria drive rapid nutrient turnover, while fungi specialize in degrading recalcitrant polymers such as cellulose and lignin and modulate algal dynamics and carbon–phosphorus cycling through saprotrophic and parasitic interactions–functional roles that are largely complementary between the two domains (Grossart et al., 2019). Traditionally regarded as predominantly terrestrial, fungi are now known–owing to high-throughput sequencing–to be widespread in diverse aquatic habitats, where they act in concert with bacteria to mediate material and energy fluxes (Chen et al., 2018; Sen et al., 2022). The FUNGuild pipeline enables functional annotation of fungal trophic guilds from sequencing data (Nguyen et al., 2016; Zheng et al., 2021). Nevertheless, systematic investigations into the seasonal succession and functional differentiation of planktonic fungi in karst river systems remain poorly understood.

Karst rivers are characterized by extensive fractures and conduits that facilitate rapid transport of pollutants and microorganisms, leading to high rates of community turnover and rendering these ecosystems particularly responsive to anthropogenic disturbance (Li et al., 2024; Wu et al., 2022). Seasonal hydrological regimes–high-flow flushing in the wet season and low-flow retention in the dry season–exert strong selective pressures on microbial assemblages. However, the relative contributions of deterministic processes (e.g., niche-based environmental filtering) versus stochastic processes (e.g., dispersal, ecological drift) to microbial community assembly along karst riverine gradients remain poorly understood (Wu et al., 2022).

Previous microbial surveys in the Chishui River basin have largely focused on the mid–lower reaches near the Moutai liquor production area, with most studies targeting bacteria and archaea (Lyu et al., 2021). In contrast, investigations into planktonic fungal community assembly within the upper Zhaotong reach remain limited. Furthermore, most existing studies adopt single-season and single-taxon sampling designs, lacking integrated comparisons of bacterial and fungal responses across wet and dry seasons. In this study, we established three sampling sites along an anthropogenic disturbance gradient: the upstream Chishuiyuan site (CSY, a lightly disturbed forested reference site), the midstream Guozhu site (GZ, subject to combined agricultural and domestic runoff inputs), and the downstream Erlongqiangbao site (EL, receiving cumulative urban pollutant inputs). Surface water samples were collected in both wet and dry seasons and analyzed using high-throughput sequencing to characterize the community structure, functional potential, and assembly processes of planktonic bacteria and fungi. We propose three core testable hypotheses: (1) total nitrogen and total phosphorus act as primary drivers shaping spatial variation in microbial communities; (2) Bacterial and fungal assemblages exhibit substantially divergent seasonal assembly trajectories, whereby deterministic homogeneous selection dominates bacteria year-round, whereas fungi are hypothesized to shift from deterministic to stochastic assembly potentially driven by dry-season hydrological and thermal stress; (3) The spatial disturbance gradient exerts dominant control over community structure, whereas seasonal hydrological variation serves as a secondary modulator. This study aims to provide scientific support for water resource protection and ecological health assessment of karst headwater rivers.

Although previous studies have investigated bacterioplankton communities in the Chishui River, they have largely relied on single-season snapshot sampling (predominantly during the wet season), with primary emphasis on bacterial diversity and compositional patterns. Consequently, the seasonal dynamics of fungal communities and the comparative assembly mechanisms between bacteria and fungi remain critically understudied. Moreover, the upper reaches of the Chishui River originate from typical karst landscapes, where groundwater–surface water interactions, carbonate-rich hydrochemistry, and pronounced seasonal hydrological fluctuations may collectively impose strong selective filters on aquatic microbial communities. Yet, the ecological implications of these karst-specific environmental constraints for microbial co-assembly have received little attention.

To address these knowledge gaps, we simultaneously investigated planktonic bacterial and fungal communities across three longitudinal sections (upper, middle, and lower reaches) of this karst-headwater river during both the dry and wet seasons. By integrating amplicon sequencing, null-model-based assembly analysis (iCAMP), and functional prediction tools, we aimed to: (i) characterize the spatiotemporal successional patterns of bacterial and fungal communities; (ii) quantitatively compare the relative contributions of deterministic versus stochastic processes governing community assembly between the two kingdoms; and (iii) assess whether their assembly dynamics respond synchronously or decoupledly to seasonal hydrological shifts. To our knowledge, this study provides the first comparative seasonal framework for bacterial–fungal co-assembly in a karst river system, offering novel insights into the differential ecological strategies of prokaryotic and eukaryotic microorganisms under fluctuating environmental filters.

## 2 Materials and methods

### 2.1 Study area and sample collection

Three sampling sites were established along the anthropogenic disturbance gradient in the Zhaotong segment of the upper Chishui River: upstream Chishuiyuan (CSY, lightly disturbed forested headwater), midstream Guozhu (GZ, affected by combined agricultural and township domestic inputs at the Yudong outlet), and downstream Erlongqiangbao Bridge (EL, a regular monitoring site receiving urban converged runoff). Surface water at a depth of 0–50 cm was sampled in January 2025 (dry season, winter) and May 2025 (wet season, spring). Three biological replicates were collected at each section, with 10 L water collected per replicate. Samples were transported to the laboratory in chilled, dark containers. Samples were temporarily stored at −20 °C, while subsamples for chemical analysis and DNA extraction were stored at −80 °C.

### 2.2 Physicochemical parameters and water quality assessment

*In situ* water temperature, pH, dissolved oxygen (DO), and conductivity were measured using a calibrated multiparameter sensor. Water samples for nutrient determination were refrigerated at 4 °C and analyzed within 24 h. Total nitrogen (TN), total phosphorus (TP), ammonium nitrogen (NH_4_^+^-N), and nitrate nitrogen (NO_3_^–^-N) were measured in accordance with Standard Methods for the Examination of Water and Wastewater (4th edition, State Environmental Protection Administration [SEPA], 2002a). Specifically, TN and TP were determined by alkaline potassium persulfate digestion; NH_4_^+^-N was analyzed via Nessler’s reagent spectrophotometry; and NO_3_^–^-N was quantified using cadmium reduction spectrophotometry.

Water quality evaluation was performed using the single-factor index method based on the Environmental Quality Standards for Surface Water (GB 3838-2002). The comprehensive water quality grade was determined by the poorest-performing single indicator among TN, TP, NH_4_^+^-N, NO_3_^–^-N, DO, and pH. Samples with TN concentrations exceeding 2.0 mg/L were classified as high-nitrogen samples, based on the Class V TN threshold in GB 3838-2002. The evaluation results were applied to identify nitrogen and phosphorus pollution hotspots and provide baseline data for subsequent microbe–environment correlation analysis (State Environmental Protection Administration [SEPA], 2002b).

### 2.3 Total DNA extraction from samples

A 600 mL water sample was pre-filtered through a 20 μm sieve to remove large particles, followed by vacuum filtration using a 0.22 μm sterile membrane filter to concentrate planktonic bacteria and fungi. The filter membranes were sectioned on a clean bench, and total DNA was extracted using the Omega Water DNA Kit (Omega, USA). DNA quality was verified via 1% agarose gel electrophoresis and UV spectrophotometry. DNA of acceptable quality showed intact bands with no signs of degradation, with an OD_260_/_280_ ratio ranging from 1.8 to 2.0 and concentrations of 6.23–26.93 ng/μL, which met the requirements for library construction and sequencing.

### 2.4 PCR amplification and high-throughput sequencing

The V3-V4 region of the bacterial 16S rRNA gene was amplified using primers 338F and 806R, while the fungal ITS region was amplified using primers ITS1F and ITS2R. Preliminary experiments were conducted to determine the optimal cycle number, and the identical low-cycle protocol was applied to all samples to minimize amplification bias. The PCR system is shown in Supplementary Table 1. Amplification was performed using TransStart Fastpfu DNA polymerase on an ABI GeneAmp^®^ 9700 PCR thermocycler. Three technical replicates were prepared for each sample, and PCR products from replicates were pooled and examined by 2% agarose gel electrophoresis. Target fragments were gel-purified using the AxyPrep DNA Gel Extraction Kit and eluted with Tris-HCl buffer. Purified DNA libraries were sequenced on an Illumina platform at Majorbio Bio-Pharm Technology Co., Ltd., (Shanghai, China).

### 2.5 Bioinformatics analysis

#### 2.5.1 Quality control and preprocessing of sequencing data

Raw sequencing reads were processed through quality control and chimera removal to obtain valid sequences. The average number of valid bacterial reads per sample was 361,292 (mean length = 418 bp), while fungal reads averaged 58,682 (mean length = 246 bp). Basic sequence preprocessing, including quality control and rarefaction, was performed using the bioinformatics analysis platform of Majorbio Bio-Pharm Technology Co., Ltd., (Shanghai, China). To minimize biases due to uneven sequencing depth, all samples were rarefied to the minimum sequencing depth. Rarefaction curves were plotted based on the amplicon sequence variant (ASV) matrix to evaluate sequencing coverage completeness.

#### 2.5.2 Community composition and alpha diversity

Relative abundances of taxa were calculated at the phylum and genus levels. Stacked bar charts of the top 10 most abundant taxa were generated to reveal spatial and seasonal variations in community composition. Observed ASVs, Shannon, Simpson, and Chao1 indices were calculated to characterize community richness and diversity. For pairwise comparisons, Student’s *t*-test, Welch’s *t*-test, or Wilcoxon rank-sum test was selected according to data distribution. For multi-group comparisons, the Kruskal-Wallis *H* test was performed first. Where significant differences were identified, Dunn’s test with false discovery rate (FDR) correction was used for *post-hoc* pairwise comparisons (see section 2.6 for details).

#### 2.5.3 Beta diversity and community assembly mechanisms

Non-metric multidimensional scaling (NMDS) ordination was performed based on the Bray–Curtis distance for visualization, and permutational multivariate analysis of variance (PERMANOVA) and analysis of similarities (ANOSIM) tests were used to examine intergroup differences in microbial communities.

The β-nearest taxon index (βNTI) and Raup–Crick (RC) indices were first applied to distinguish deterministic and stochastic assembly processes. Pairwise comparisons with —βNTI| ≥ 2 were classified as deterministic selection: βNTI > 2 indicates homogeneous selection (HoS), while βNTI < −2 indicates heterogeneous selection (HeS). Pairwise comparisons with —βNTI| < 2 were classified as stochastic processes, which were further partitioned using the RC index: dispersal limitation (DL, RC > 0.95) and homogenizing dispersal (HD, RC < −0.95).

The iCAMP framework was then used to quantify the relative contributions of five assembly processes: HoS, HeS, DL, HD, and drift (DR). Only ASVs with prevalence ≥ 0.1 were retained for phylogenetic binning to minimize noise from rare taxa.

#### 2.5.4 Taxonomic differences and functional potential prediction

In the genus-level abundance matrix, taxa with relative abundance < 20 were grouped as “Others,” while unclassified genera were retained separately. The simplified matrix was used to generate stacked bar charts. The linear discriminant analysis effect size (LEfSe) method (LDA threshold ≥ 2.0, FDR-corrected *p* < 0.05) was used to identify indicator taxa across multiple taxonomic levels, and the results were visualized via cladograms and LDA bar plots (Segata et al., 2011).

Functional profiles of bacteria were predicted using PICRUSt2. ASVs were aligned against the GTDB release 207 reference tree, and phylogenetic placement was implemented via EPA-NG. After copy number normalization, functional information was aggregated into Kyoto Encyclopedia of Genes and Genomes (KEGG) Level 2 pathways, with emphasis on carbon, nitrogen, phosphorus metabolism and environmental adaptation pathways (Douglas et al., 2020).

Fungal functional annotation was performed using FUNGuild based on the UNITE v8.3 database. Only taxa with confidence scores ≥ 80% were retained, and the abundances of trophic modes including saprotroph, pathogen and symbiotroph were quantified (Nguyen et al., 2016).

#### 2.5.5 Community–environment association analysis

Eight physicochemical variables were selected: water temperature, pH, DO, conductivity, TN, TP, NH_4_^+^–N, and NO_3_^–^–N. Pearson correlation analysis showed severe multicollinearity among variables (—r| > 0.85, *p* < 0.01, VIF > 20). Variables with VIF > 10 were removed; pH, TP, and NH_4_^+^–N were retained for the wet-season redundancy analysis (RDA), whereas pH and TP were retained for the dry-season RDA. Detailed VIF values are provided in Supplementary Table 2.

Prior to ordination, the species matrix was Hellinger-transformed and environmental variables were standardized. The significance of each environmental variable was assessed via 999 permutation tests (*p* < 0.05). Following the filtering criteria used in iCAMP analysis, only ASVs with prevalence ≥ 0.1 were included to minimize interference from rare ASVs.

### 2.6 Data processing and statistical analysis

All statistical analyses were performed using R v4.x.x. For multi-group comparisons of environmental variables, one-way Analysis of Variance (ANOVA) was used for datasets conforming to normality and homogeneity of variance; otherwise, the Kruskal-Wallis H test was applied. PERMANOVA based on Bray-Curtis distance with 999 permutations was used to evaluate differences in community structure.

Multiple comparison tests (e.g., Dunn’s test, LEfSe) were corrected using the Benjamini-Hochberg FDR method, with corrected *p* < 0.05 considered significant. Single permutation tests (PERMANOVA, ANOSIM, RDA) were evaluated directly using raw *p* < 0.05. In this manuscript, *p*-values marked “FDR” are corrected values; unmarked values are raw *p*-values. All results are presented as mean ± standard deviation (Mean ± SD).

## 3 Results

### 3.1 Spatiotemporal characteristics of aquatic physicochemical parameters

Physicochemical indicators were measured at the three sampling sites (CSY, GZ, EL) in the Zhaotong reach of the Chishui River during January 2025 (dry season) and May 2025 (wet season) (Table 1). Spatially, concentrations of TN, TP, and dissolved inorganic nitrogen (DIN) increased continuously from CSY to GZ and EL, whereas DO decreased downstream, forming an inverse gradient. Meanwhile, pH and conductivity increased in parallel, yielding a consistent spatial pattern of low-disturbance upstream, transitional midstream, and nutrient-enriched downstream conditions.

**TABLE 1:** Physicochemical parameters of water in the Zhaotong reach of the Chishui River in spring and winter.

Site	Season	Temp. (C)	pH	DO (mg/L)	Cond (μS/cm)	TN (mg/L)	TP (mg/L)	NH_4_^+^-N (mg/L)	NO_3_^–^-N (mg/L)
CSY	Spring	16.8 ± 0.3	7.42 ± 0.05	9.15 ± 0.12	182 ± 5	1.24 ± 0.08	0.04 ± 0.01	0.12 ± 0.02	2.64 ± 0.05
CSY	Winter	7.8 ± 0.3	7.54 ± 0.05	10.65 ± 0.12	209.3 ± 5.75	0.84 ± 0.05	0.03 ± 0.01	0.08 ± 0.01	1.48 ± 0.03
GZ	Spring	17.5 ± 0.4	7.58 ± 0.07	8.63 ± 0.09	196 ± 7	1.86 ± 0.11	0.07 ± 0.01	0.18 ± 0.03	2.92 ± 0.07
GZ	Winter	8.5 ± 0.4	7.70 ± 0.07	10.13 ± 0.09	225.4 ± 8.05	1.26 ± 0.07	0.05 ± 0.01	0.12 ± 0.02	1.92 ± 0.05
EL	Spring	18.4 ± 0.3	7.86 ± 0.04	7.92 ± 0.10	231 ± 6	2.87 ± 0.16	0.12 ± 0.02	0.35 ± 0.05	4.56 ± 0.11
EL	Winter	9.4 ± 0.3	7.98 ± 0.04	9.42 ± 0.10	265.65 ± 6.90	2.15 ± 0.12	0.09 ± 0.02	0.26 ± 0.04	3.17 ± 0.08

Seasonally, water temperature was lower in the dry season (7.8–9.4 °C) than in the wet season (16.8–18.4 °C). DO concentrations were markedly higher in the dry season due to greater O_2_ solubility at lower temperatures. Nutrient concentrations were generally higher in the wet season, suggesting enhanced non-point source inputs during rainfall. According to GB 3838-2002, DO, pH, and NH_4_^+^-N complied with Class II or better. TN increased from Class I at CSY to worse than Class V at EL (2.87 mg/L), and TP reached Class III at EL (0.12 mg/L), indicating notable downstream accumulation of nitrogen and phosphorus.

### 3.2 Structure and functional prediction of planktonic bacterial communities

Given this distinct downstream gradient of nutrients and water quality, we performed high-throughput amplicon sequencing to systematically compare the diversity, community structure and assembly mechanisms of planktonic bacterial and fungal assemblages.

After quality control and rarefaction, an average of 25,297 valid reads were obtained for spring samples and 23,297 for winter samples. Sequencing coverage exceeded 97% across all samples (Table 2). The rarefaction curves gradually plateaued with increasing sequencing depth, confirming adequate sequencing depth to capture the taxonomic information of planktonic bacteria in the water column. These data formed a reliable dataset for subsequent analyses of diversity and community structure.

**TABLE 2:** Alpha diversity indices of planktonic bacteria.

Samples	Observed ASVs	Shannon index	Simpson index	Ace index	Chao1 index	Coverage/%
Spring						
CSY-S	2564 ± 278a	6.18 ± 0.28a	0.0123 ± 0.0052a	2784 ± 361a	2643 ± 351a	98.64 ± 0a
GZ-S	1236 ± 225b	4.83 ± 0.12b	0.0341 ± 0.0057bc	1290 ± 289b	1255 ± 251b	99.60 ± 0b
EL-S	1056 ± 125b	4.41 ± 0.09b	0.0481 ± 0.0030b	1093 ± 151b	1067 ± 135b	99.68 ± 0b
Winter						
CSY-W	2121 ± 72a	5.84 ± 0.01a	0.0233 ± 0.0011a	2291 ± 94a	2330 ± 70a	97.65 ± 0.08a
GZ-W	1041 ± 109b	4.66 ± 0.12b	0.0345 ± 0.0034b	1145 ± 151b	1136 ± 148b	98.29 ± 0.22b
EL-W	865 ± 123b	4.02 ± 0.19b	0.0743 ± 0.0143b	924 ± 153b	1073 ± 160b	98.36 ± 0.16b

The lowercase letters (a, b) represent the results of the significance test. Within the same column, data with different lowercase letters indicate significant differences between samples (*P* < 0.05), while data with the same letter indicate no significant difference (*P* > 0.05).

The observed coverage values were consistent with expectations for amplicon sequencing: bacterial 16S coverage ranged from 97% to 99%, whereas fungal ITS coverage was generally higher ( > 99%). This reflects inherent differences between the two marker genes in primer universality and the conservation of target regions. Overall, the richness and diversity of planktonic bacterial communities were higher in spring than in winter, showing clear seasonal patterns. Furthermore, the spatial differentiation characteristics changed markedly among seasons.

#### 3.2.1 Sequencing depth and alpha diversity analysis

Rarefaction curves (Supplementary Figure 1) plateaued gradually, indicating sufficient sequencing depth. Kruskal–Wallis H tests conducted at the ASV level revealed no statistically significant differences in the Ace index among the three sampling sites in spring (*p* = 0.5611). This suggests strong river connectivity during the wet season, and no significant spatial differentiation in bacterial species richness was detected between upstream and downstream reaches. Numerically, upstream CSY spring samples had the highest observed ASVs, Chao1, and Shannon indices, and the lowest Simpson index. This implies that the clean upstream water supported higher species richness and community evenness, although such differences were not statistically significant.

In the dry season, the Ace index differed significantly among the three sections (Kruskal-Wallis H test, *p* < 0.001). *Post-hoc* pairwise comparisons (Dunn’s test with FDR correction) demonstrated that the Ace index of CSY-W was significantly higher than those of GZ-W and EL-W (corrected *p* < 0.05), whereas no significant difference existed between GZ-W and EL-W. Richness decreased in the order CSY-W > GZ-W > EL-W. Reduced runoff and intensified habitat isolation in winter strengthened environmental filtering, leading to significant divergence in species richness between upstream and downstream sites.

Paired seasonal Wilcoxon tests at each sampling site showed that observed ASVs, Chao1, and Shannon indices at upstream CSY were significantly lower in winter than in spring, accompanied by an increase in the Simpson index. This indicates that low winter temperature exerted particularly strong inhibitory effects on bacterial communities at CSY. The proportion of dominant taxa increased, and the overall community evenness declined.

#### 3.2.2 Spatial and seasonal differentiation of planktonic bacterial communities

NMDS ordination and Unweighted Pair-Group Method with Arithmetic Mean (UPGMA) hierarchical clustering were performed based on the Bray-Curtis distance matrix to visualize spatial clustering patterns of planktonic bacterial communities in spring and winter (Figure 1).

**FIGURE 1:**
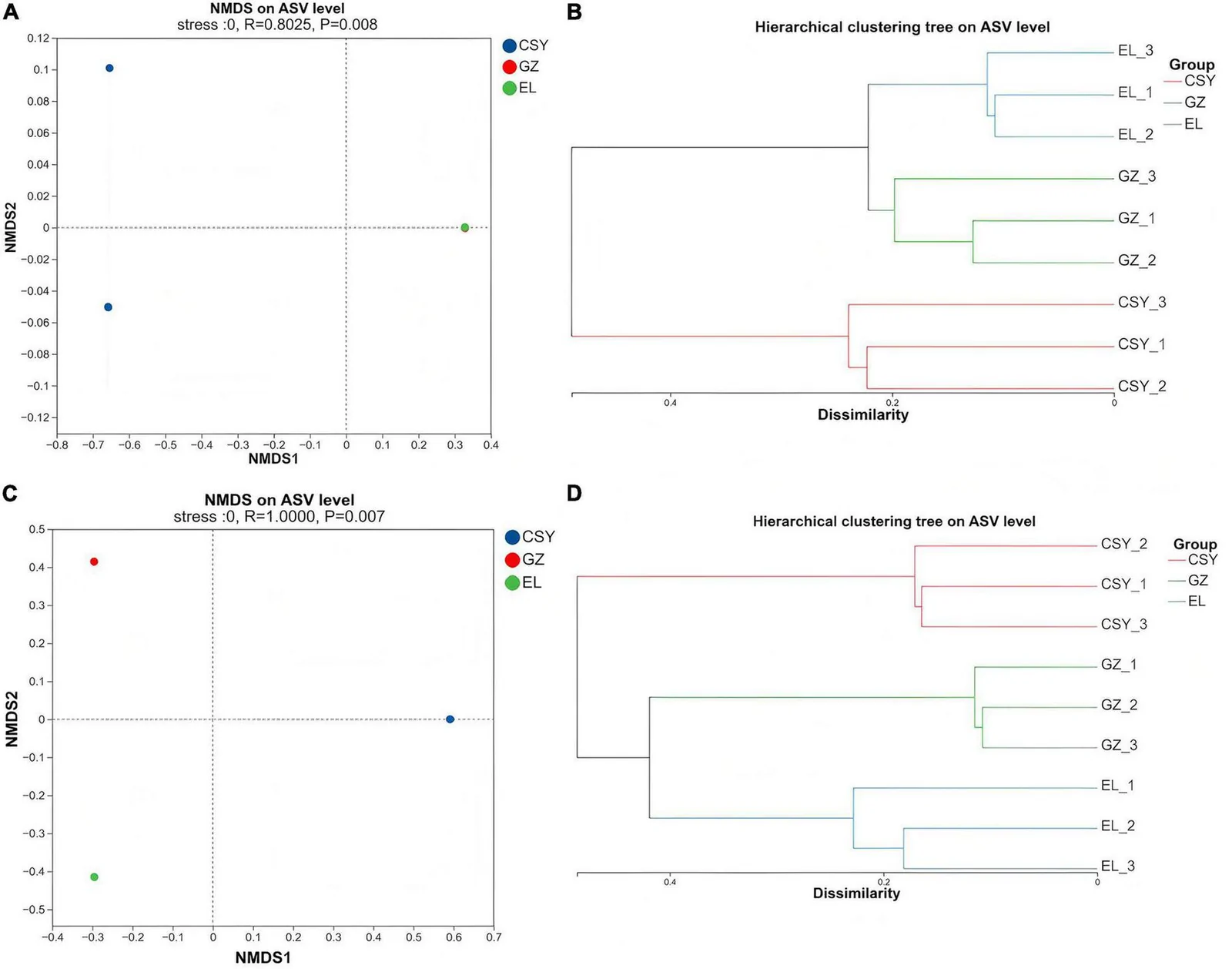
Non-metric multidimensional scaling (NMDS) ordination **(A,C)** and UPGMA hierarchical clustering dendrograms **(B,D)** showing spatial and seasonal patterns of planktonic bacterial communities. **(A,B)** Wet season (spring). **(C,D)** Dry season (winter). Each point represents an individual sample, colored by sampling site (CSY, upstream; GZ, midstream; EL, downstream). Ellipses indicate 95% confidence intervals. Branch lengths of UPGMA trees correspond to dissimilarity among samples. PERMANOVA results are shown in the panels.

Spring NMDS ordination (Figure 1A) showed a good model fit (Stress = 0, *R*^2^ = 0.8025), and PERMANOVA confirmed significant intergroup differences (*p* = 0.008). Samples formed three clusters corresponding to sampling sites: CSY-S separated independently on the left, while GZ-S and EL-S were distributed on the right with partial overlap, indicating moderate similarity between midstream and downstream communities. The UPGMA clustering tree (Figure 1B) further supported this pattern: CSY-S formed a distinct branch, and GZ-S and EL-S clustered into one group.

Both winter NMDS ordination (Figure 1C) and UPGMA clustering tree (Figure 1D) revealed clear separation among the three sample groups. However, the ordination distance between CSY and the other two sites (GZ, EL) decreased relative to spring, and the branching distance between GZ and EL was shortened, reflecting enhanced homogenization of midstream and downstream communities. PERMANOVA confirmed that sampling site had a significant effect (*R*^2^ = 0.935, *p* = 0.006). Compared with spring, midstream and downstream communities were more homogenized in winter, and the spatial disparity between upstream and downstream was narrowed.

#### 3.2.3 Assembly mechanisms of planktonic bacterial communities

To elucidate the ecological processes underlying the observed spatiotemporal patterns, we applied the iCAMP framework to quantify the relative contributions of five assembly processes: HoS, HeS, DL, HD, and DR (Figure 2). In the wet season, HoS and HD jointly governed community assembly along the entire river reach. HoS accounted for 35%–46% of assembly events, indicating that, despite high hydrological connectivity, spatially uniform water chemistry exerted consistent selective pressure. HD contributed 25%–30%, promoting community similarity among sites, while DL and DR together explained 21%–23%, maintaining measurable spatial turnover. HeS was negligible ( < 1%), reflecting limited environmental heterogeneity across the reach. This combination of processes generated a wet-season clustering pattern, wherein communities exhibited partial but incomplete spatial differentiation. In the dry season, HoS became the dominant assembly process (40%–65%). Low temperature and reduced discharge constrained niche availability and metabolic activity, thereby strengthening homogeneous environmental filtering. Deterministic processes thus prevailed, whereas stochastic dispersal played a diminished role. The relative importance of assembly processes also varied along the anthropogenic disturbance gradient. At the upstream site CSY, the highest HoS contribution (∼65%) likely arises from the combined influences of dense forest vegetation, low pollutant inputs, and stable karst water chemistry. Forested catchments generally deliver relatively uniform allochthonous organic substrates. Meanwhile, limited human disturbance sustains consistent nutrient stoichiometry and carbonate-buffered pH. Collectively, these background environmental conditions impose strong spatially homogeneous selection on planktonic microbes, which strengthens deterministic community assembly. By comparison, more variable agricultural and urban pollutant inputs at GZ and EL break this homogeneous environmental filtering, giving stochastic processes greater relative importance. We note that other unmeasured local-scale variables may also contribute to this pattern. At the midstream site GZ, agricultural non-point source inputs increased microhabitat heterogeneity, leading to a moderate decline in HoS and a corresponding increase in DL. At the downstream site EL, high hydrological connectivity and flow variability reduced the dominance of local environmental filtering, enabling DR to increase markedly and act in conjunction with HoS to shape community structure.

**FIGURE 2:**
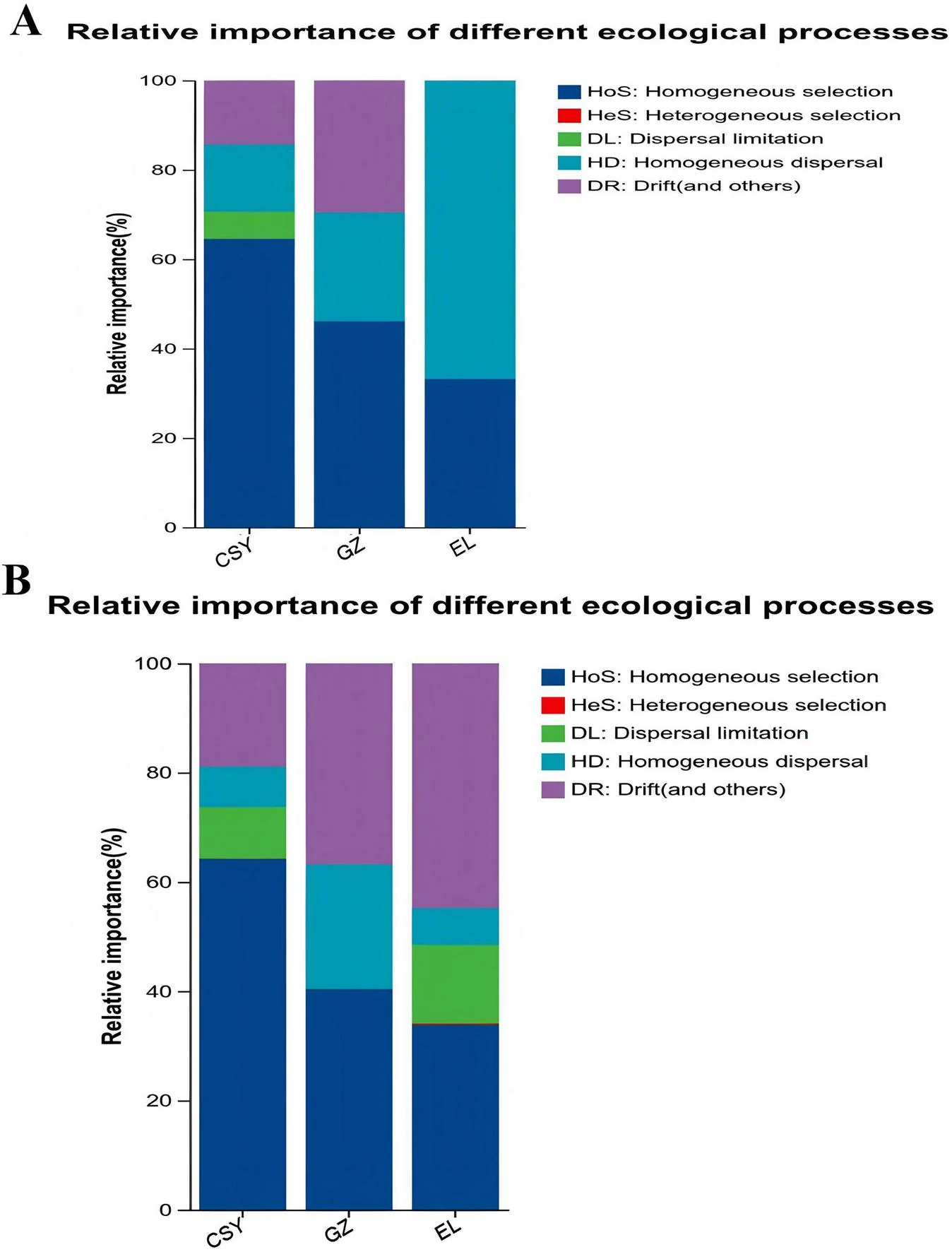
Relative contributions of ecological processes to bacterial community assembly in the wet **(A)** and dry **(B)** seasons, quantified via iCAMP. HoS, HeS, DL, HD, DR. Percentages indicate the relative contribution of each process at each site.

Collectively, deterministic homogeneous selection consistently governed bacterial assembly across both hydrological seasons, with stochastic processes only playing secondary roles along the disturbance gradient.

#### 3.2.4 Analysis of community composition and dominant taxa of planktonic bacteria

Stacked bar charts of genus-level relative abundances revealed pronounced seasonal turnover and longitudinal spatial differentiation of dominant planktonic bacterial taxa (Figure 3A for spring; Figure 3B for winter).

**FIGURE 3:**
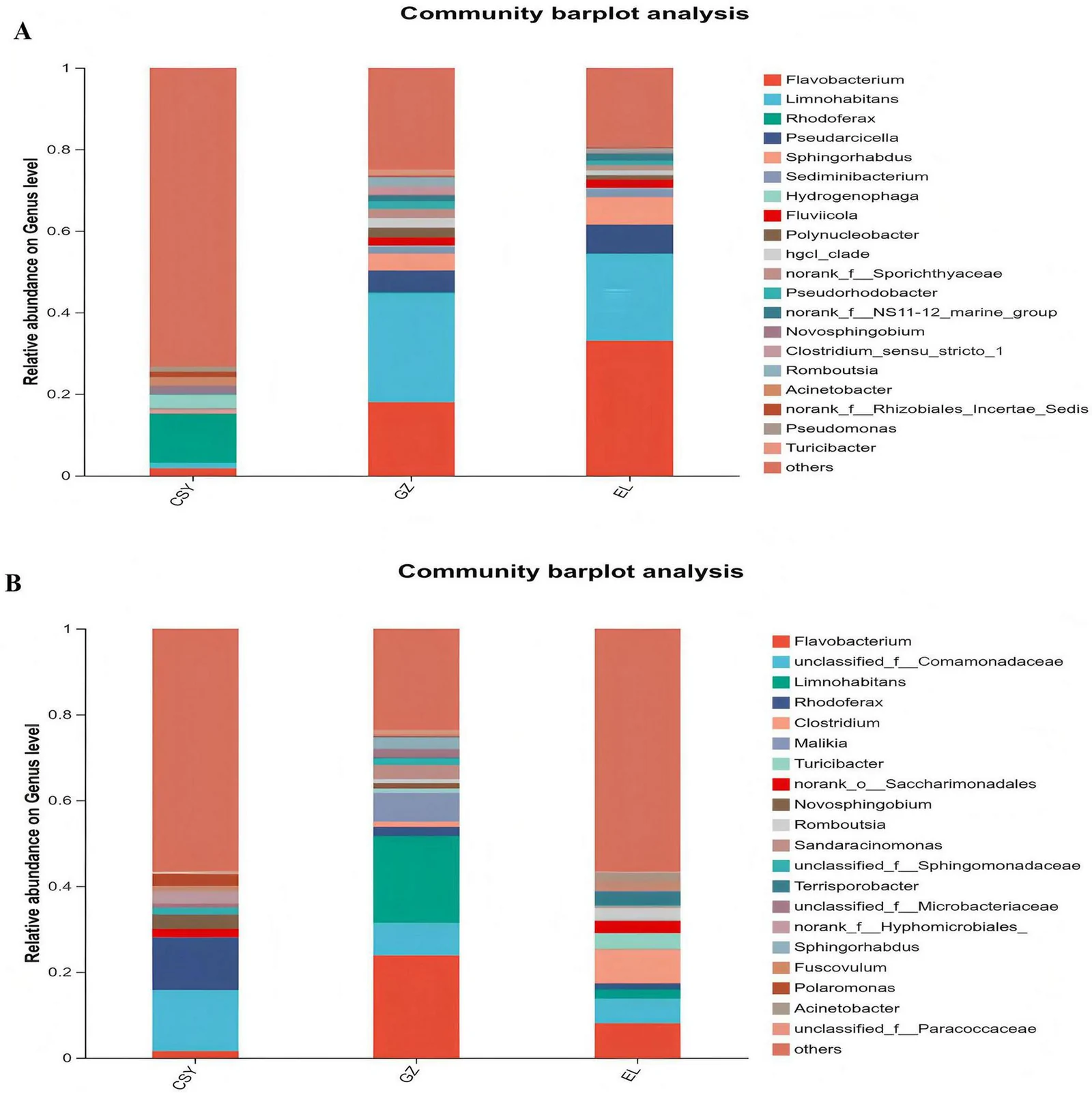
Genus-level relative abundances of planktonic bacteria in the wet season (A, spring) and dry season (B, winter). Taxa with low abundance are not shown. LEfSe biomarkers for each season are indicated (see Supplementary Figures 1, 2 for cladograms).

In spring, *Flavobacterium* was overwhelmingly the dominant genus at upstream CSY, with its relative abundance exceeding 95%, while other genera accounted for only minor proportions. Community structures became more complex at midstream GZ and downstream EL; the abundance of *Flavobacterium* decreased to 30%–45%, whereas the proportion of *Limnohabitans* increased to 20%–30%. Associated taxa including *Rhodoferax* and *Pseudarcicella* were consistently detected.

Community composition underwent substantial restructuring in winter. The dominance of Flavobacterium declined at upstream CSY, accompanied by an increased proportion of unclassified taxa. A complete shift in dominant taxa occurred at midstream GZ: Limnohabitans exceeded 50% and replaced Flavobacterium as the primary dominant genus. At downstream EL, Flavobacterium remained one of the dominant genera (∼30–35%). Meanwhile, cold-tolerant and anaerobic bacteria such as Clostridium_sensu_stricto_1 (∼15%) and Malikia (∼10%) were enriched, resulting in higher community complexity compared with upstream and midstream sites.

LEfSe analysis further identified season-specific indicator taxa (LDA ≥ 2.0, FDR-corrected *p* < 0.05). Spring biomarkers all belonged to *Flavobacterium* (phylum Bacteroidota). Winter indicator taxa were mainly represented by *Limnohabitans* (Proteobacteria), alongside several taxa affiliated with Actinobacteriota. The complete LEfSe cladograms and LDA bar plots for spring and winter are provided in Supplementary Figures 2, 3, respectively.

#### 3.2.5 Functional potential of planktonic bacterial communities predicted by PICRUSt2

Functional gene profiles were predicted from 16S rRNA gene sequences using PICRUSt2. Predicted functional profiles were analyzed at the KEGG Level 2 hierarchy to assess variations in the potential metabolic capabilities of planktonic bacterial communities (Figure 4).

**FIGURE 4:**
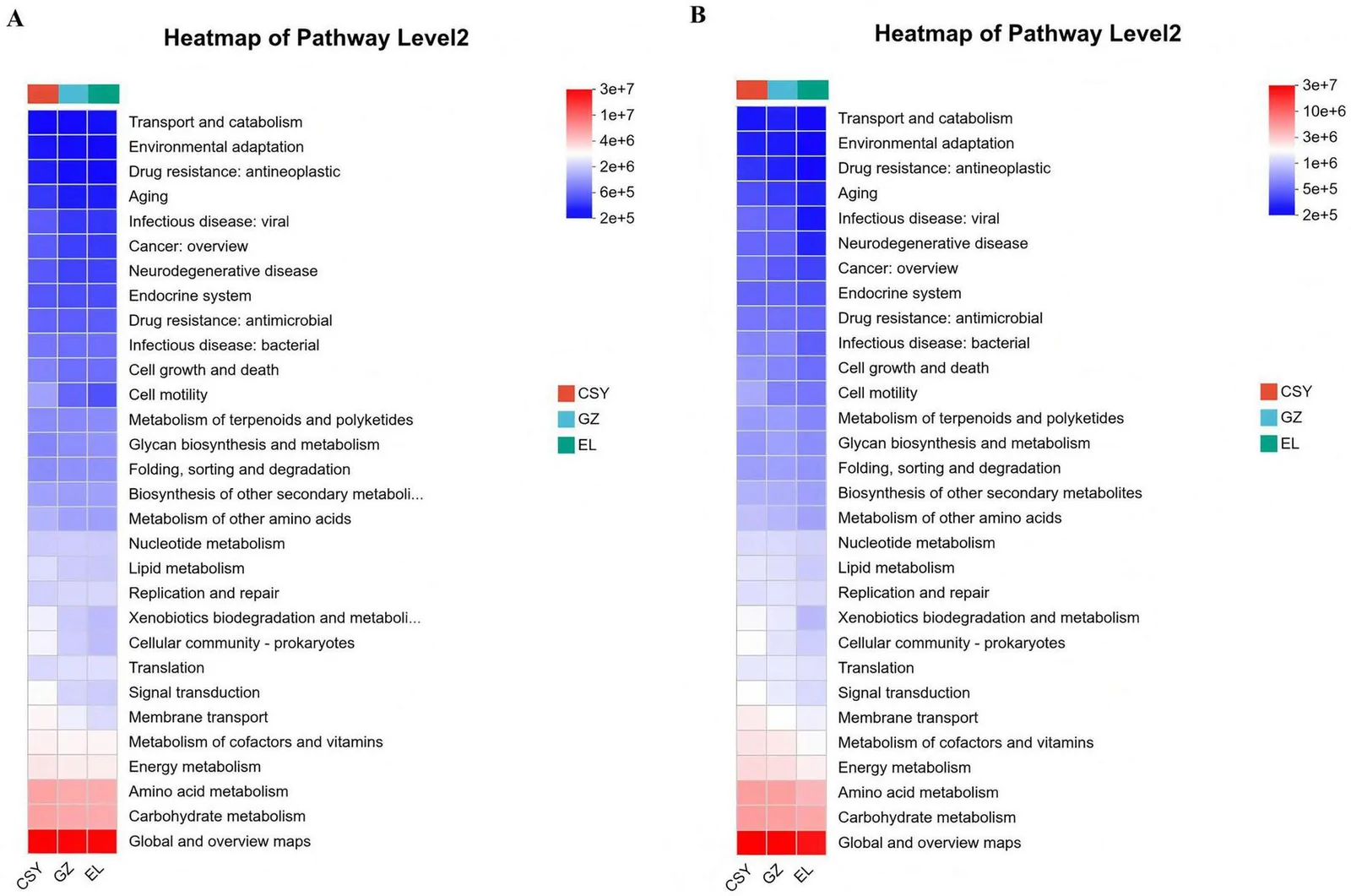
Heatmaps of predicted KEGG Level 2 functional profiles for planktonic bacteria based on PICRUSt2. **(A)** Wet season (spring). **(B)** Dry season (winter). CSY, upstream; GZ, midstream; EL, downstream. The color gradient corresponds to predicted gene copy numbers.

In the wet season, predicted relative abundances of pathways related to environmental adaptation, cellular stress response, and virulence-associated functions were consistently high across all sections, whereas core metabolic pathways–including carbohydrate and amino acid metabolism–exhibited comparatively lower predicted relative abundances (Figure 4A). Spatially, predicted gene copy numbers associated with cell motility and signal transduction were elevated in upstream CSY samples. Midstream GZ showed higher predicted relative abundances of terpenoid and polyketide secondary metabolite biosynthesis pathways. Downstream EL was characterized by enriched predicted pathways involved in protein folding, sorting, and degradation, possibly reflecting heightened protein homeostasis demands under urban-influenced conditions.

In the dry season, predicted functional profiles shifted overall, with increased relative abundances of fundamental metabolic pathways, including carbohydrate metabolism, amino acid metabolism, and energy metabolism (Figure 4B). Both CSY and GZ were dominated by core metabolic pathways, and inter-site differences were modest, primarily manifesting in membrane transport-related functions.

#### 3.2.6 Correlation between planktonic bacterial communities and environmental factors

Pearson correlation analyses among eight physicochemical variables revealed severe multicollinearity (—r| > 0.85, VIF > 20); consequently, only pH, TP and NH_4_^+^-N were retained for RDA in the wet season, whereas only pH and TP were retained in the dry season. In the wet season, the first two RDA axes explained 37.66% of the total constrained variation in bacterial community composition (RDA1: 28.36%, RDA2: 9.30%). Both TP and pH were significant explanatory variables, with relatively long vectors in the RDA biplot, whereas the NH_4_^+^-N vector was shorter, indicating a weaker explanatory power (Figure 5A).

**FIGURE 5:**
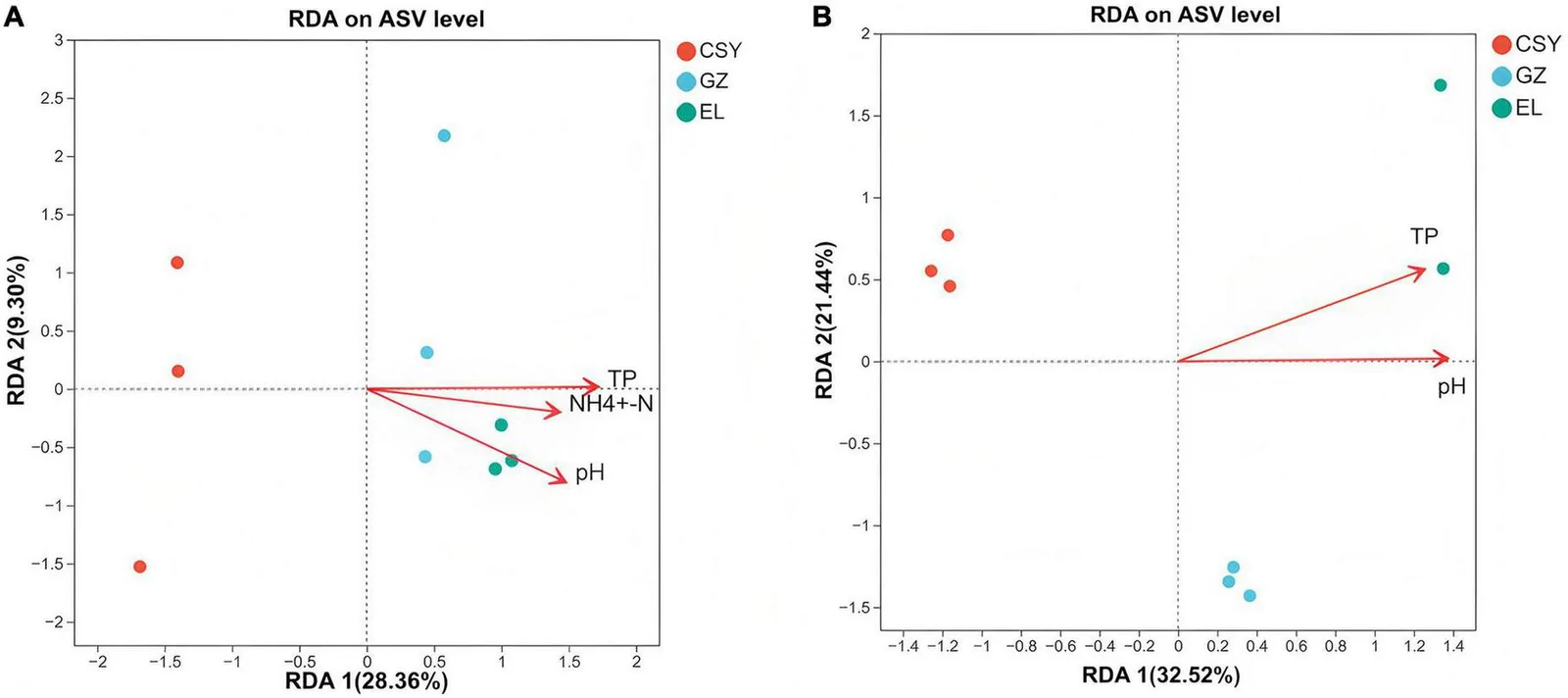
Redundancy analysis (RDA) illustrating relationships between planktonic bacterial communities and environmental variables (TN, TP) in the wet **(A)** and dry **(B)** seasons. Points denote individual samples, colored by sampling site (CSY, upstream; GZ, midstream; EL, downstream). Arrows represent environmental vectors for TN and TP. The fit and *p*-values for each variable, as well as the explained variation for each RDA axis, are shown in the panels.

In the wet season, samples separated clearly along the RDA1 axis: CSY clustered in the low-pH and low-TP quadrant, whereas GZ and EL grouped within the high-pH and high-TP quadrant (Figure 5A). This spatial partitioning persisted into the dry season, where the first two RDA axes explained 53.96% of the total constrained variation (RDA1: 32.52%, RDA2: 21.44%). CSY still clustered in the low-pH and low-TP quadrant, while GZ and EL were distributed in the high-pH and high-TP region (Figure 5B). Collectively, these findings identify pH and TP as key environmental variables associated with spatial variation in planktonic bacterial communities.

#### 3.2.7 Alpha diversity of planktonic fungi

The rarefaction curves (Supplementary Figure 4) plateaued gradually, indicating sufficient sequencing depth. Table 3 and Figures 3, 4 summarize ASV-level alpha diversity of planktonic fungi. In spring, the Ace index did not differ significantly among the three sites (Kruskal-Wallis *H* test, *p* = 0.05091), suggesting strong river connectivity during the wet season and limited spatial differentiation in fungal richness. In winter, the Ace index varied significantly across sections (*p* = 0.02732). *Post-ho*c pairwise comparisons (Dunn’s test with FDR correction) showed that CSY-W (median ≈ 1580) had significantly higher Ace values than GZ-W (median ≈ 1040) and EL-W (median ≈ 1000), with no difference between GZ-W and EL-W. This pattern indicates stronger environmental filtering in winter, with the upstream site maintaining higher fungal diversity.

**TABLE 3:** Alpha diversity indices of planktonic fungi in spring and winter.

Samples	Sobs index	Shannon index	Simpson index	Ace index	Chao1 index	Coverage/%
CSY-S	1465 ± 105	5.55 ± 0.01	0.0162 ± 0.0004a	1486 ± 119	1479 ± 115	99.87 ± 0
GZ-S	985 ± 61	5.07 ± 0.06	0.0238 ± 0.0058a	1001 ± 61	994 ± 61	99.90 ± 0
EL-S	941 ± 64	5.39 ± 0.11	0.0106 ± 0.0008a	945 ± 64	943 ± 64	99.97 ± 0
CSY-W	1422.33 ± 39.19a	4.94 ± 0.03	0.025 ± 0.002a	1626.76 ± 169.21a	1675.65 ± 175.02a	99.90 ± 0.0011b
GZ-W	1231.00 ± 243.94b	5.14 ± 0.12	0.028 ± 0.004a	1283.70 ± 266.68b	1293.28 ± 306.42b	99.66 ± 0.0029a
EL-W	1084.00 ± 149.50b	4.48 ± 0.16	0.052 ± 0.017a	1179.22 ± 209.43b	1202.94 ± 215.83b	99.51 ± 0.0023a

Data are presented as mean ± standard deviation. Different lowercase letters (a, b) within the same column indicate significant differences among sites within the same season (Kruskal-Wallis H test followed by Dunn’s test with FDR correction, *P* < 0.05). No letters indicate no significant difference among sites. Seasonal differences between spring and winter were assessed using paired Wilcoxon tests and are not denoted by letters in this table.

Seasonally, the Ace index at GZ and EL increased by approximately 28% and 25% in winter relative to spring, and the absolute Ace differences between CSY and the two downstream sites narrowed (485 → 343 for CSY vs GZ; 541 → 448 for CSY vs EL).

Paired seasonal Wilcoxon tests revealed that the Shannon index at CSY was significantly lower in winter than in spring (*p* < 0.05), with a concurrent increase in the Simpson index. At GZ, the Shannon index rose slightly from 5.07 to 5.14, whereas at EL it declined from 5.39 to 4.48, with the Simpson index increasing from 0.0106 to 0.052, indicating reduced evenness and enhanced dominance of specific taxa at the downstream site.

#### 3.2.8 Spatial and seasonal differentiation of planktonic fungal communities

NMDS ordination and UPGMA hierarchical clustering were performed based on Bray–Curtis dissimilarities at the ASV level. NMDS yielded low stress values (wet season: stress < 0.001; dry season: stress < 0.001), confirming a reliable representation of community relationships. ANOSIM further revealed strong and statistically significant separation among sampling sites (wet season: Global *R* = 0.944, *p* = 0.003; dry season: Global *R* = 0.972, *p* = 0.004), indicating that among-group differences substantially exceeded within-group variation (Figure 6). Sampling site was the dominant factor structuring fungal communities, with all samples clustering strictly according to the three sections (CSY, GZ, and EL). In both seasons, the upstream CSY community formed a distinct cluster separate from midstream and downstream groups, whereas GZ and EL assemblages converged into a single cluster. Notably, the increase in species richness and decline in community evenness at GZ and EL during the dry season coincided with a reduced branching distance between these sites in the UPGMA dendrogram, reflecting decreased β-diversity and heightened compositional similarity. Collectively, these findings demonstrate that planktonic fungal communities maintained a stable spatial configuration characterized by a distinctive upstream assemblage and convergent midstream–downstream communities. Seasonal hydrological fluctuations primarily reshaped species abundance distributions but did not override the overarching spatial gradient in fungal community structure.

**FIGURE 6:**
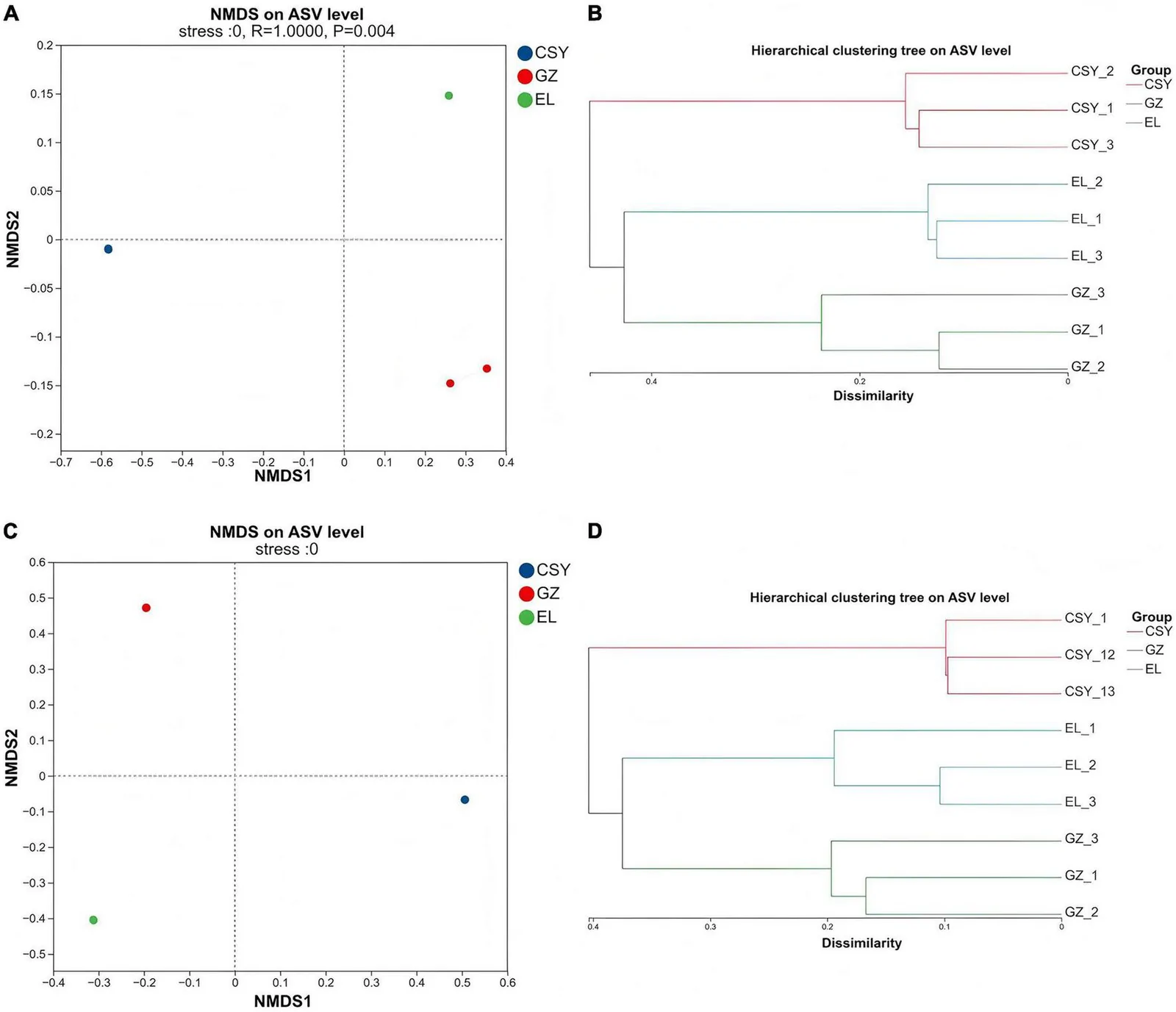
Non-metric multidimensional scaling (NMDS) ordination **(A,C)** and UPGMA hierarchical clustering dendrograms **(B,D)** of planktonic fungal communities based on Bray-Curtis dissimilarities at the ASV level. **(A,B)** Wet season (spring). **(C,D)** Dry season (winter). Each point represents an individual sample, colored by sampling site (CSY, upstream; GZ, midstream; EL, downstream). Branch lengths in UPGMA trees correspond to community dissimilarity. ANOSIM statistics (Global R and *p*-values) are shown in the panels.

#### 3.2.9 Assembly mechanisms of planktonic fungal communities

The iCAMP framework was used to quantify the relative contributions of five community assembly processes (Figure 7).

**FIGURE 7:**
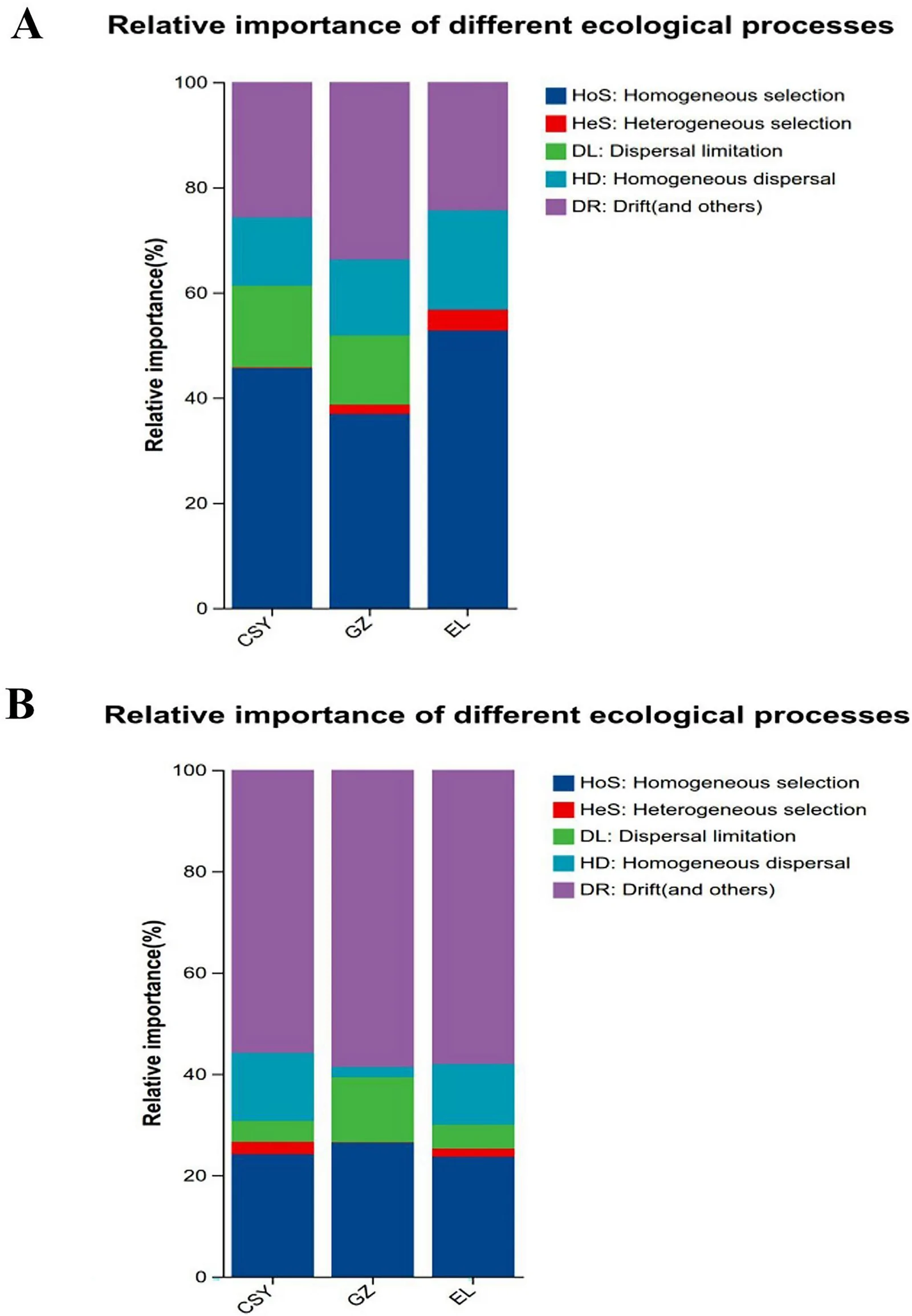
Relative contributions of ecological processes governing planktonic fungal community assembly in the wet **(A)** and dry **(B)** seasons, quantified via iCAMP. HoS, homogeneous selection; HeS, heterogeneous selection; DL, dispersal limitation; HD, homogenizing dispersal; DR, drift. Percentages indicate the relative contribution of each process at each site (CSY, upstream; GZ, midstream; EL, downstream).

In the wet season, the contribution of HoS ranged from 44% to 46% along the whole river reach, reaching 46% at upstream CSY (Figure 7A). The proportions of DL, HD and DR were relatively low, while HeS contributed negligibly. This indicates that uniform aquatic environmental filtering served as the primary driver structuring fungal communities during the wet season.

In the dry season, the contribution of HoS decreased sharply to 10%–14%, and stochastic processes became predominant (Figure 7B). The proportion of DR increased to 46%–50%, accompanied by a synchronous rise of HD contribution above 30%. The weakened environmental filtering effect in this season strengthened the regulation of community structure by stochastic dispersal and extinction, coinciding with community homogenization at midstream GZ and downstream EL.

Overall, deterministic homogeneous selection dominated fungal community assembly in the wet season, whereas stochastic processes including ecological drift and homogenizing dispersal jointly governed community assembly in the dry season.

In stark contrast to persistent deterministic filtering in bacterial communities, fungal assembly underwent a full seasonal transition from homogeneous selection to stochastic drift-dominated assembly.

#### 3.2.10 Seasonal dynamics and spatial heterogeneity of planktonic fungal community composition and dominant taxa

Genus-level stacked bar charts and LEfSe multi-level difference analysis illustrated pronounced spatiotemporal turnover in fungal assemblages (Figure 8). A clear spatial gradient emerged along the river continuum. In the wet season, the upstream CSY site was enriched in Ascomycota genera with terrestrial saprotrophic and plant-pathogenic affinities, including *Didymella*, *Cercospora*, *Cladosporium*, and *Boeremia* (Figure 8A). During the dry season, basidiomycetous genera *Epicoleosporium* and *Mycodiella* increased in relative abundance, and low-abundance taxa grouped as “others” constituted a noticeable proportion of the community (Figure 8B). At midstream GZ, unclassified Fungi (kingdom-level assignments) dominated in the wet season ( > 50% relative abundance), while terrestrial fungi were scarce. Community composition shifted markedly in the dry season: *Cladosporium* increased to approximately 20%, whereas unclassified Fungi declined correspondingly. The downstream EL site was consistently enriched in Rozellomycota lineages across both seasons, representing a distinctive aquatic fungal signature unique to this reach.

**FIGURE 8:**
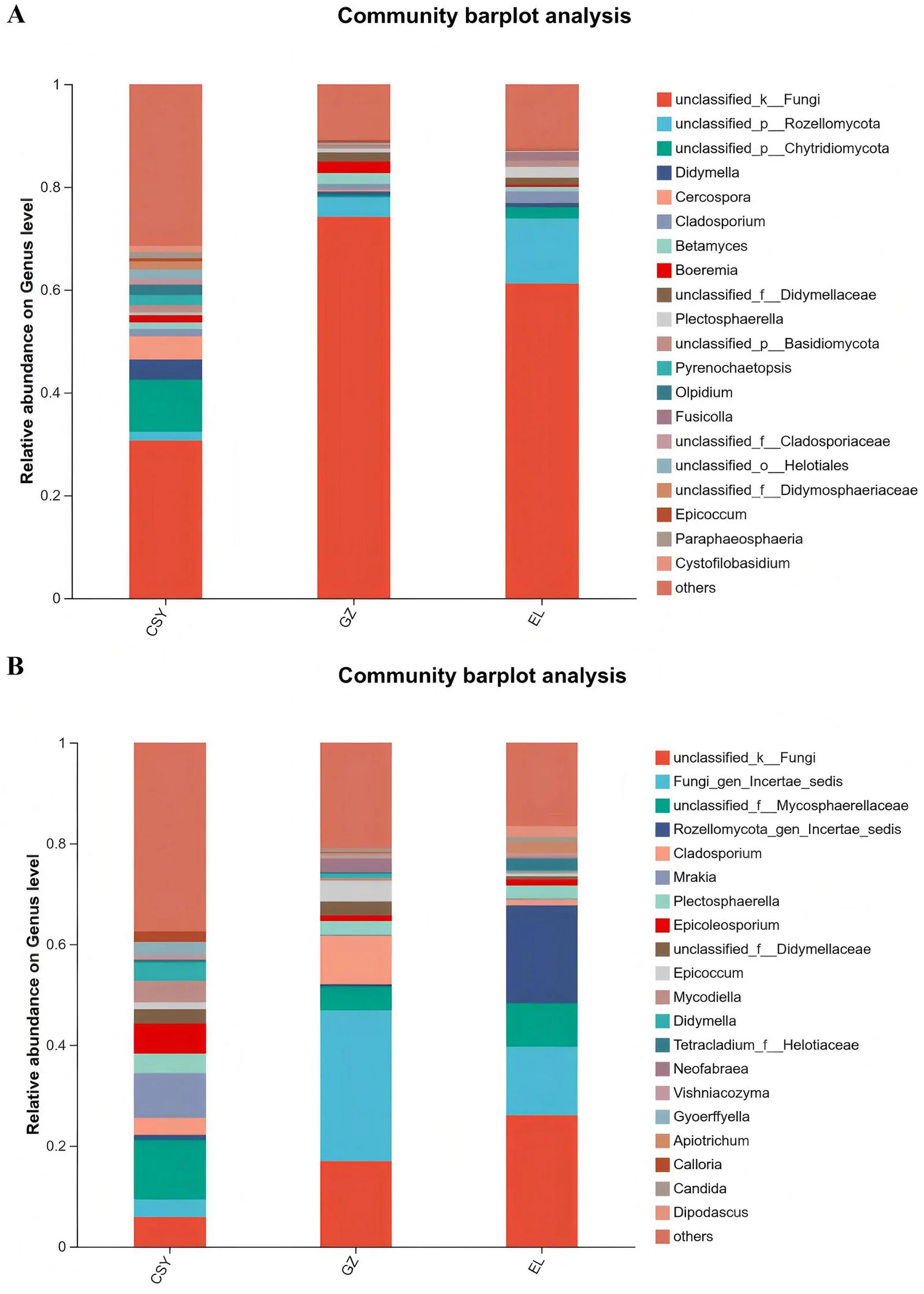
Stacked bar charts showing the genus-level relative abundance of planktonic fungal communities. **(A)** Wet season (spring). **(B)** Dry season (winter). CSY, upstream; GZ, midstream; EL, downstream. Each colored segment denotes the relative abundance of a single fungal genus; low-abundance taxa are grouped as “others.”

Dominant taxa displayed clear seasonal turnover across all sites. At CSY, *Didymella* prevailed in the wet season, whereas *Epicoleosporiu*m and *Mycodiella* became more prominent in the dry season. At GZ, terrestrial fungi were sparse during the wet season but *Cladosporium* proliferated in the dry season. At EL, unclassified Fungi and Rozellomycota dominated in the wet season, while the terrestrial pathogenic fungus *Cercospora* increased noticeably in the dry season.

LEfSe analysis further identified season-specific indicator taxa (Supplementary Figures 5, 6; LDA ≥ 2.0, FDR-corrected *p* < 0.05). Spring biomarkers were primarily associated with Ascomycota lineages enriched at the upstream CSY site, whereas winter biomarkers were predominantly affiliated with Basidiomycota and Rozellomycota at midstream and downstream sites.

#### 3.2.11 Functional group composition of planktonic fungi

Fungal trophic modes were annotated using FUNGuild against the UNITE v8.3 database, retaining assignments with a confidence score ≥ 80%. Pronounced spatiotemporal variation was evident in the functional structure of planktonic fungal communities (Figure 9).

**FIGURE 9:**
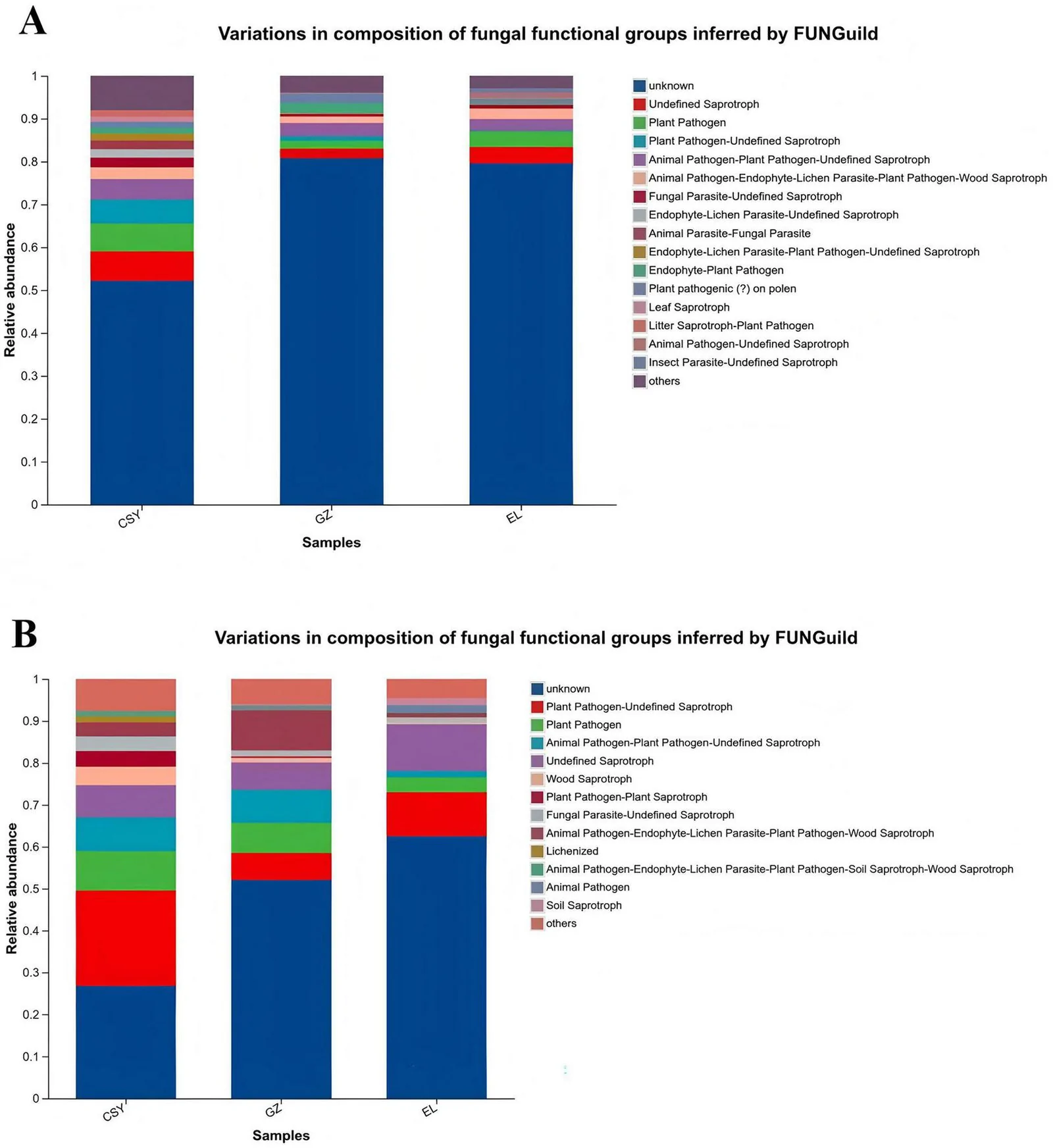
Stacked bar charts showing relative abundances of fungal trophic functional groups inferred via FUNGuild. **(A)** Wet season (spring). **(B)** Dry season (winter). CSY, upstream; GZ, midstream; EL, downstream. Low-abundance functional groups are grouped as “others.”

In the wet season, the diversity of identified trophic guilds was limited across all sites (Figure 9A). Taxa with unresolved trophic status (“Unknown”) and those annotated as plant pathogens or saprotrophs with unspecified substrate affinity together accounted for approximately 80% of total read abundance. The relative abundance of “Unknown” taxa followed the order GZ > EL > CSY, while the combined plant pathogen–undefined saprotroph guild also peaked at CSY. Other trophic modes were present only at low relative abundances.

In the dry season, the richness of identified trophic guilds increased markedly (Figure 9B). Upstream CSY retained a simple functional profile dominated by “Unknown” taxa and plant pathogens. Midstream GZ was enriched in generalist saprotrophic guilds capable of utilizing animal, plant, and wood substrates. Downstream EL exhibited a substantial increase in undefined saprotrophs, alongside concurrent enrichments of animal pathogens, endophytes, and saprotrophs. Among all samples, EL displayed the highest functional group richness in the dry season.

Overall, fungal functional composition appeared largely homogeneous across the river reach in the wet season, primarily reflecting the prevalence of taxa with unresolved trophic status. In the dry season, the diversity of trophic modes expanded considerably at midstream and downstream sites, resulting in clearer spatial differentiation in fungal functional structure.

#### 3.2.12 Correlation between planktonic fungal communities and environmental factors

RDA was conducted based on the ASV abundance matrix to examine relationships between fungal communities and the environmental variables retained after collinearity filtering (Figure 10).

**FIGURE 10:**
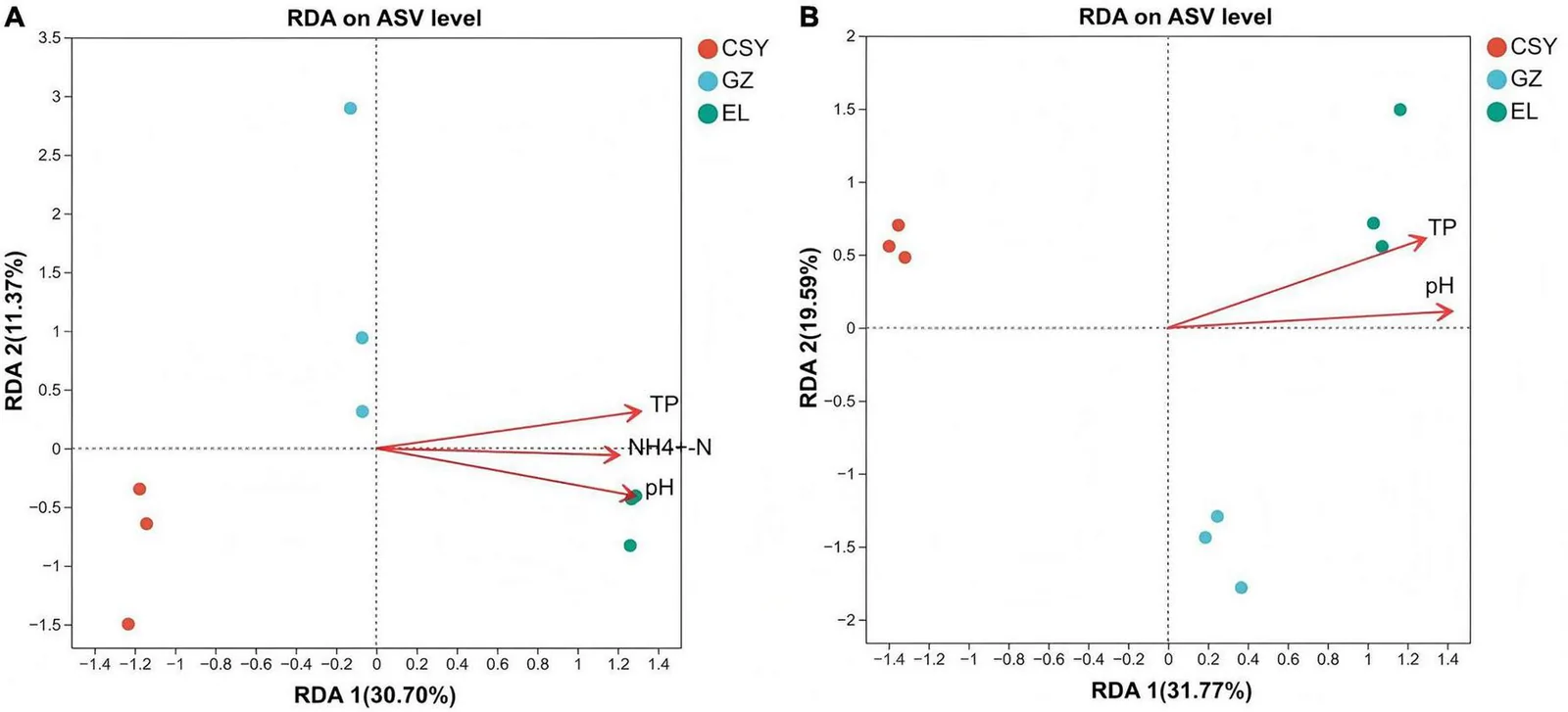
Redundancy analysis (RDA) illustrating relationships between planktonic fungal communities (ASV level) and environmental variables (TN, TP) in the wet **(A)** and dry **(B)** seasons. Points represent individual samples, colored by sampling site (CSY, upstream; GZ, midstream; EL, downstream). Arrows denote environmental vectors for TN and TP. The explained variation for each RDA axis is shown in the panels.

In the wet season, the first two RDA axes explained 30.70% and 11.37% of the constrained variation, respectively (cumulative = 42.07%) (Figure 10A). TP, NH_4_^+^–N, and pH all showed positive correlations with RDA1 (*r* > 0.90). Permutation tests confirmed that TP (fit = 89.6%, *p* = 0.004) and pH were significant explanatory variables. Samples segregated along the nutrient gradient: CSY clustered in the low-nutrient, low-pH quadrant (negative RDA1 axis), whereas GZ and EL grouped within the high-nutrient, high-pH range.

In the dry season, RDA1 and RDA2 accounted for 31.77% and 19.59% of the constrained variation, respectively (cumulative = 51.36%) (Figure 10B). The vectors of TP and pH pointed toward the positive RDA1 axis; accordingly, CSY clustered in the negative quadrant, while GZ and EL shifted to the positive quadrant. This contrasting ordination pattern suggests that the relationship between fungal community structure and the TP-pH gradient shifts with season, possibly reflecting altered hydrological regimes.

Overall, these results indicate that TP and pH are key environmental variables correlated with spatial variation in planktonic fungal communities, with a stronger association in the dry season.

## 4 Discussion

### 4.1 Spatiotemporal patterns of aquatic physicochemical factors

Longitudinal variations in water physicochemical variables showed consistent trends in both seasons. TN, TP and inorganic nitrogen increased from upstream to downstream, while DO declined gradually. This spatial pattern persisted across seasons, with seasonal shifts only altering concentration magnitudes rather than reversing the upstream–downstream gradient. Such spatial differentiation closely matched the anthropogenic disturbance intensity along the river.

The karst dual hydrological structure provides the geological foundation for this stable nutrient gradient. Carbonate rock outcrops create strong surface water–groundwater connectivity, allowing rapid pollutant transport via conduits and sinkholes (Wu et al., 2022; He et al., 2011). Pollutants are not fully degraded during transport, and rainfall runoff can convey non-point pollutants into groundwater and river channels within short periods (Wang et al., 2025). Long-term monitoring data from the Chishui River support this pattern: Zhou et al. (2021) observed increasing permanganate index and ammonia nitrogen in the midstream, and Teng et al. (2016) confirmed agricultural runoff as the primary nutrient source. These karst transport characteristics weaken natural self-purification and preserve spatial concentration differences from anthropogenic pollution, forming a persistent nutrient gradient.

RDA results from this study confirmed TP and pH as core drivers of bacterial community variation, and TN and TP as core drivers of fungal community variation (*p* < 0.05), with ordination plots clearly separating upstream low-nutrient from midstream and downstream high-nutrient samples. Comparable patterns have been documented in diverse ecosystems: Logue et al. (2012) found nutrient availability as the primary controller of bacterial richness in oligotrophic lakes, and Hu et al. (2022) confirmed nitrogen and phosphorus gradients shaping bacterial spatial differentiation in the Yangtze River basin. Seasonal variations do not reverse the spatial nutrient ranking but modulate environmental filtering intensity via water temperature and discharge–a framework that provides the environmental context for seasonal divergence in bacterial and fungal assembly.

### 4.2 Seasonal decoupling in community assembly mechanisms of bacteria and fungi

Quantitative iCAMP analyses revealed a stark seasonal divergence in how planktonic bacteria and fungi assemble. In spring, HoS dominated both groups, yet the underlying filters differed markedly. Intensive rainfall flattened physicochemical gradients along the reach, locking total nitrogen and phosphorus into a steady downstream decline (Wu et al., 2022; Zhou et al., 2021; Wang et al., 2024). Fast-growing bacteria, sustained by large population sizes, appeared to compete for the same nutrient pools at every site–likely explaining the resource-driven HoS observed here (Simon, 1988; Newton et al., 2011). Fungi followed a different trajectory: runoff delivered massive loads of terrestrial spores into the channel. Because most of these taxa could at least persist in water, local habitat differences exerted weak sorting pressure. Consequently, fungi also exhibited HoS, but this pattern arose independently of nutrient filtering. By winter, these assembly pathways diverged substantially. Deterministic HoS persisted for bacteria, whereas DR governed fungal assemblages (Figures 3, 9), pointing to taxon-specific sensitivities to hydrological and thermal stress.

Such contrasting pathways are seldom documented in karst headwaters, where surface and groundwater exchange is intense. Most studies to date focus on single taxonomic groups or overlook karst systems entirely. The upper Chishui reach, characterized by pronounced seasonal hydrological swings superimposed on a persistent nutrient gradient, appears to push bacteria and fungi onto separate assembly tracks. This divergence likely reflects the combined influence of local karst geology, winter temperature regimes, and anthropogenic disturbances–factors that argue for the simultaneous monitoring of both groups in vulnerable karst aquatic ecosystems. Spatial patterns further shifted along the disturbance gradient. Upstream pristine sites exhibited the strongest HoS signals. At GZ, however, agricultural inputs increased microhabitat heterogeneity and made community assembly more stochastic. In the dry season, reduced hydrological connectivity associated with lower runoff likely constrained inter-site dispersal of fungal spores, potentially intensifying the relative influence of ecological drift. This pattern is consistent with the expectation that diminished water exchange under low-flow conditions facilitates stochastic community assembly.

The seasonal flip in filtering intensity likely reflects divergent abiotic constraints. Wet-season mixing positioned resource competition as the primary selective force. In winter, declining temperatures probably suppressed microbial metabolism, shifting the filter toward cold tolerance. Fungi, reliant on spore-dependent life cycles, appear particularly susceptible to dry-season stress. Low temperatures can hinder spore germination and hydrolase synthesis, which may shrink metabolically active fungal populations and magnify drift effects (Zhou and Ning, 2017). Homogenizing dispersal (HD) rates collapsed to merely 2%–13% in the dry season, which could limit allochthonous fungal propagule supply, while DR accounted for 46%–50% of fungal assembly variation–surpassing the 45% maximum observed for bacteria. Notably, even elevated nutrient levels at the downstream EL site failed to override this stochastic dominance. This suggests that thermal and hydrological conditions may outweigh nutrient filtering for fungi, whereas bacterial assembly remained tethered to nutrient availability year-round.

These trends mirror those reported in other river systems showing bacteria adhering to deterministic processes while fungi shift toward stochasticity under harsh conditions (Hao et al., 2025; Jia et al., 2024; Zhang et al., 2022; Xu et al., 2024). For example, in the Dali River, a non-karst tributary of the Yellow River on the Loess Plateau, Chen et al. (2025) similarly found that sediment bacterial communities were governed by deterministic processes, whereas fungal communities were dominated by stochastic processes. However, the Dali River is characterized by loess-derived surface runoff and soil erosion, whereas our study system is a karst headwater stream with strong surface-groundwater connectivity and rapid conduit-based pollutant transport (Wu et al., 2022). These karst-specific hydrological traits–particularly the ‘pulse-isolation’regime of wet-season flushing versus dry-season disconnection–may amplify seasonal contrasts in dispersal and drift for spore-dispersing fungi. We therefore caution that the decoupled assembly pattern observed here may be system-specific and not directly generalizable to non-karst fluvial systems without further comparative studies. The split probably stems from fundamental life-history disparities: bacteria maintain substantial active populations at low temperatures, allowing continued exploitation of nutrient niches, whereas fungal dispersal and reproductive output are severely constrained by cold, low-flow conditions. We acknowledge that direct physiological thresholds were not measured here and warrant verification via microcosm experiments. Nonetheless, this life-history contrast offers a parsimonious explanation for the complete seasonal transition in fungal assembly–from HoS dominance to drift-driven stochasticity–while bacterial communities retained consistent, nutrient-regulated selection across both seasons.

### 4.3 Divergent patterns of indicator taxa and functional groups along the disturbance gradient

This kingdom-specific decoupling in assembly processes should, in turn, leave distinct imprints on the spatiotemporal distributions of dominant lineages–a pattern we next examined across the disturbance gradient by tracking indicator taxa and trophic guilds. *Flavobacterium* dominated the upstream CSY site in spring, reaching > 95% relative abundance–far above background levels typical of most rivers–and likely representing ecotypes adapted to oligotrophic headwaters (Cottrell and Kirchman, 2000). Its abundance dropped sharply downstream as substrate diversity increased. Notably, *Flavobacterium* persisted at EL during winter, often co-occurring with cold-tolerant genera such as *Clostridium_sensu_stricto_1*. This suggests the genus encompasses physiologically distinct lineages rather than being strictly confined to pristine habitats. LEfSe analysis supported strong seasonal taxon turnover: spring biomarkers aligned with *Flavobacterium* (Bacteroidota), whereas winter indicators shifted toward *Limnohabitans* (Proteobacteria), tracking seasonal changes in substrate availability. The spring enrichment of Flavobacterium at the upstream site aligns with its well-documented oligotrophic lifestyle. Members of this genus carry high-affinity substrate uptake systems and can utilize a wide range of dissolved organic compounds even at low ambient concentrations (Cottrell and Kirchman, 2000; Newton et al., 2011). In contrast, Limnohabitans is commonly found in moderately eutrophic freshwaters and responds rapidly to pulsed substrate inputs (Newton et al., 2011). Its higher relative abundance in winter may therefore be linked to lower-flow conditions that potentially concentrate available resources, although this hydrological mechanism remains to be directly tested. Such pronounced fluctuations imply that bacterial assemblages remain under year-round, nutrient-driven homogeneous selection. Consequently, *Flavobacterium* may be more useful for tracing short-term runoff pulses than for flagging chronic anthropogenic disturbance.

In contrast, fungal indicator taxa showed greater temporal stability. Continuous forest runoff supplied terrestrial spores and humus to upstream sites, sustaining Ascomycota, Basidiomycota, and Blastocladiomycota, along with persistent terrestrial plant pathogens such as *Cercospora*, *Cladosporium*, and *Boeremia* (Pham et al., 2024). Agricultural and urban effluents at midstream GZ markedly reduced terrestrial fungal loads; here, unclassified fungi prevailed and rare taxa diminished (Lynch and Neufeld, 2015). Downstream at EL, Rozellomycota remained consistently enriched across both seasons. As obligate parasites of algae and zooplankton (Lara et al., 2010), Rozellomycota appeared relatively insensitive to the seasonal stochasticity that otherwise governs fungal assembly. This seasonal stability carries clear ecological implications. As obligate parasites, Rozellomycota rely on suitable planktonic hosts to survive (Lara et al., 2010). Based on existing ecological theory, host biomass is predicted to rise under nutrient-enriched conditions, although host abundance was not quantified in our dataset. Their persistent enrichment at downstream EL, where TN and TP reach the highest levels across our sampling sites, therefore likely points to an indirect trophic linkage with nutrient-stimulated host proliferation, instead of a direct response to nutrient concentrations *per se*. Their steady presence, coupled with tolerance of elevated nutrients, positions them as potential long-term indicators of anthropogenic nutrient enrichment–offering a contrast to nutrient-sensitive bacterial taxa such as *Flavobacterium*.

Fungal trophic guilds also shifted seasonally. FUNGuild annotations revealed that unknown fungi combined with plant pathogen–undefined saprotrophs accounted for roughly 80% of reads in spring, yielding functionally homogenized communities. In winter, longer water residence times allowed exogenous organic matter to accumulate, promoting diverse saprotrophic guilds at GZ and undefined saprotrophs at EL, thereby increasing the functional diversity predicted by FUNGuild (Grossart et al., 2019). These shifts are likely associated with changing inputs of allochthonous organic matter, although we emphasize that FUNGuild annotations reflect metabolic potential rather than realized in-situ activities, and direct linkages to dissolved and particulate organic carbon measurements remain to be tested.

The high proportion of taxa with unresolved trophic status (“Unknown”) in the wet season partly reflects current limitations in freshwater fungal databases, particularly for early-diverging lineages and environmental clones (Ishida et al., 2015). This underscores the need for improved functional annotation of aquatic fungi in future studies.

### 4.4 Ecological indicator value and research limitations

Our synthesis of community assembly patterns, taxonomic biomarkers, and fungal trophic guilds points to several potential indicators of anthropogenic disturbance in karst headwater streams. At the assembly level, fungal ecological drift exceeding 55% in winter–coupled with weakened homogeneous selection–appears to track sustained human pressure. Taxonomically, we observed a clear longitudinal gradient: intact terrestrial fungal assemblages upstream, simplified communities at midstream GZ, and seasonally stable enrichment of Rozellomycota downstream. Among these, *Rozellomycota* emerges as a preliminary candidate cross-seasonal indicator, although its utility requires further validation across independent field datasets. As obligate parasites of phytoplankton and zooplankton, Rozellomycota have been increasingly recognized as responsive to nutrient-driven shifts in aquatic food webs (Lara et al., 2010). Functionally, elevated fungal trophic diversity during the dry season at GZ and EL coincided with increased allochthonous organic inputs. Bacteria and fungi also diverged in indicator behavior: *Flavobacterium* tracked short-term nutrient pulses, whereas Rozellomycota responded more consistently to longer-term pollution gradients. These candidates, however, now need to be tested against independent datasets before any operational use. We therefore stress that these candidate bioindicators should be regarded as preliminary hypotheses instead of fully validated biomarkers. Their broader applicability needs systematic field-based validation across multiple karst headwater river systems.

A major limitation is that we lack direct, high-frequency in-situ hydrological measurements. Although we interpret the seasonal shift in fungal community assembly processes as partly driven by flow reduction, this interpretation rests on the untested assumption that lower discharge intensifies dispersal-related constraints for fungal propagules. In reality, water temperature and water residence time strongly co-vary with discharge across dry-wet seasonal transitions, which means our current dataset cannot disentangle their independent effects on fungal assembly. Future work should deploy continuous flow and temperature loggers, paired with residence-time monitoring where feasible, to quantitatively disentangle their relative forcing on fungal community dynamics in these karst headwater systems.

Our three sampling sites represent a discrete disturbance gradient along the upper Chishui headwater reach: CSY serves as a minimally disturbed forested reference site; GZ receives mixed agricultural and domestic runoff; and EL is an urban-influenced site that accumulates pollutants from the broader catchment. Water-quality measurements corroborate this gradient: concentrations of TN, TP, and dissolved inorganic nitrogen (DIN) are significantly higher at EL than at GZ, with TN at EL exceeding the Class V threshold specified in GB 3838-2002. However, the absence of intermediate transects between CSY and GZ, as well as between GZ and EL, limits our ability to assess continuous community responses along the disturbance gradient. Moreover, the partial overlap in pollution sources between GZ and EL may introduce confounding effects when interpreting microbial community patterns. Future investigations should incorporate denser longitudinal sampling with additional intermediate sites to better resolve fine-scale disturbance–response relationships in this karst headwater stream.

Sampling was restricted to two hydrologically distinct seasons, precluding detection of transitional thresholds in assembly-process shifts. we cannot verify whether low dry-season runoff directly constrains spore dispersal and amplifies drift–a central premise of our interpretation. Functional inferences from PICRUSt2 and FUNGuild describe metabolic potential rather than in situ activity (Sun et al., 2020). By analysing bacteria and fungi separately, we also leave interkingdom interactions unexplored (Faust and Raes, 2012). Finally, the lack of quantitative dissolved and particulate organic matter data limits our ability to mechanistically interpret seasonal turnover in fungal trophic guilds.

Addressing these gaps will require more controlled approaches. Microcosm experiments, for example, could help tease apart how temperature, hydrological connectivity, and nutrient gradients differentially shape bacterial and fungal assembly, providing a direct test of the mechanisms proposed here. Extending sampling into transitional hydrological periods–ideally at a monthly resolution–would make it easier to pinpoint the temperature or discharge thresholds at which fungal assembly flips from homogeneous selection to drift. Integrating high-frequency hydrological monitoring, DOM/POM characterization, and metatranscriptomics would further ground these patterns in process-based understanding (Luo et al., 2025), moving beyond correlation toward mechanistic insight in karst river ecosystems.

## 5 Conclusion

This study focused on the karst headwater reach of the Chishui River in Zhaotong. Three sampling sections were established along an anthropogenic disturbance gradient, and synchronous sampling was conducted during the wet season (spring) and dry season (winter). Integrating 16S and ITS high-throughput sequencing with community null modeling and functional predictions, we systematically characterized divergent responses of planktonic bacteria and fungi to nutrient gradients and seasonal hydrological regimes. The main conclusions are as follows:

Concentrations of TN and TP showed a consistent spatial gradient, increasing significantly from upstream to downstream. The karst dual hydrological structure may limit the natural attenuation of pollutants along the flow path. TP and pH emerged as core environmental variables correlated with spatial variation in bacterial communities, while TN and TP were the core variables for fungal communities; seasonal hydrological fluctuations altered nutrient concentrations without reversing this upstream-downstream gradient.

Species richness of planktonic bacteria and fungi peaked at the forested upstream site across both seasons, whereas diversity declined at midstream and downstream sites influenced by agricultural and urban inputs. In the dry season, species richness increased markedly at midstream and downstream sites, accompanied by a decline in community evenness. NMDS and ANOSIM confirmed that the anthropogenic disturbance gradient was the primary driver of spatial variation in both microbial communities, with midstream and downstream assemblages becoming increasingly similar.

Assembly processes of planktonic bacteria and fungi exhibited clear seasonal divergence. Bacterial communities were consistently governed by HoS, with stochastic processes gaining relative importance along the disturbance gradient. Fungal communities were also structured by HoS in the wet season, but DR dominated in the dry season. The spore-based dispersal strategy of fungi likely contributes to their greater sensitivity to low-temperature and low-discharge stress, whereas the short generation times and large population sizes of bacteria represent a plausible mechanistic basis for their divergent assembly strategies.

Distinct spatial and seasonal patterns were observed for dominant taxa and functional potential. In the wet season, *Flavobacterium* was enriched in oligotrophic upstream waters, while *Limnohabitans* increased with nutrient levels at midstream and downstream sites. Rozellomycota fungi were consistently enriched in the polluted downstream reach. Functional predictions generated by PICRUSt2 and FUNGuild indicated that predicted metabolic pathways in the wet season were biased toward environmental adaptation, while core metabolic pathways rose in relative abundance in the dry season. Note that these tools infer gene content instead of measuring real-world metabolic rates. The richness of composite fungal trophic guilds increased at the midstream and downstream sites during the dry season, pointing to a potential yet-indirect association with allochthonous organic-matter inputs.

Planktonic bacteria and fungi exhibited distinct indicator potential. *Flavobacterium* abundance showed pronounced seasonal fluctuations, making it suitable for monitoring short-term environmental perturbations. In contrast, Rozellomycota fungi (consistently enriched downstream) and composite fungal trophic guilds (enhanced in dry-season midstream/downstream reaches) showed seasonal stability and may serve as candidate bioindicators for long-term anthropogenic pollution gradients in karst rivers.

In summary, planktonic bacteria and fungi in karst headwater rivers display taxon-specific responses to anthropogenic pollution and seasonal hydrology across community assembly, taxonomic composition, and functional dimensions. These findings advance our understanding of microbial succession and its drivers in karst systems, with implications for aquatic ecological assessment of the Chishui River–including water source protection for sauce-flavor liquor production and conservation of endemic fishes–as well as non-point source pollution control in karst mountain regions. Nevertheless, this study was limited to fixed-site sampling across two hydrologically contrasting seasons and lacked continuous hydrological monitoring and in situ functional measurements, which should be addressed in future work to strengthen the generalizability of our conclusions.

While our study focuses on a single karst headwater system, the contrasting assembly regimes of bacteria and fungi likely apply broadly to seasonally pulsed freshwater ecosystems with strong hydrological connectivity.

## Data Availability

The raw sequence data presented in this study are available in the Genome Sequence Archive (GSA) (https://ngdc.cncb.ac.cn/gsa), under accession number CRA050683.

## Supplementary material

Supplementary Figure 1 Alpha diversity and sequencing depth of planktonic bacterial communities. **(A)** Rarefaction curves in the wet season (spring). **(B)** Rarefaction curves in the dry season (winter). **(C)** ACE index boxplots in the wet season (spring). **(D)** ACE index boxplots in the dry season (winter). Colors represent sampling sites: red, CSY (upstream); blue, GZ (midstream); green, EL (downstream). Different lowercase letters (a, b) above boxplots indicate significant differences among groups (Dunn’s test, FDR-corrected **p* < 0.05); ns, non-significant.

Supplementary Figure 2 LEfSe cladogram showing bacterial indicator taxa for spring (wet season) in the upper Chishui River. The circular cladogram presents the bacterial taxonomic hierarchy from phylum to genus level. Taxa with LDA score ≥ 2.0 and FDR-corrected *P* < 0.05 were identified as spring-specific biomarkers. Spring-enriched bacterial indicators are predominantly affiliated with the genus *Flavobacterium* within the phylum Bacteroidota. Yellow nodes represent taxa without significant differential enrichment.

Supplementary Figure 3 LEfSe cladogram showing bacterial indicator taxa for winter (dry season) in the upper Chishui River. The circular cladogram displays bacterial taxonomic ranks from phylum to genus. Taxa satisfying LDA score ≥ 2.0 and FDR-corrected *P* < 0.05 correspond to winter-specific biomarkers. Winter-enriched indicators are mainly represented by *Limnohabitans* (Proteobacteria) and multiple taxa belonging to Actinobacteriota. Yellow nodes indicate taxa showing no significant differential enrichment.

Supplementary Figure 4 Rarefaction curves and Ace index-based alpha diversity analysis of planktonic fungi at the ASV level. **(A, B)** Rarefaction curves for planktonic fungi in spring **(A)** and winter **(B)**. Curves represent the sequencing coverage index against the number of sampled reads for the CSY, GZ, and EL sites. **(C,D)** Boxplots showing the Ace index of planktonic fungi in spring **(C)** and winter **(D)**, with statistical results from the Kruskal-Wallis H test. Significant differences are marked with asterisks.

Supplementary Figure 5 LEfSe cladogram showing spring-specific indicator taxa of planktonic fungi in the upper Chishui River. The circular cladogram displays the fungal taxonomic hierarchy from phylum to genus level. Taxa with LDA score ≥ 2.0 and FDR-corrected *P* < 0.05 were screened as spring-specific fungal biomarkers. Red, blue, and green branches represent the upstream CSY, midstream GZ, and downstream EL sites, respectively. Most spring-enriched fungal indicators belong to Ascomycota, with numerous differential taxa detected at the upstream CSY site. Yellow nodes indicate taxa with no significant differential enrichment.

Supplementary Figure 6 LEfSe cladogram showing winter-specific indicator taxa of planktonic fungi in the upper Chishui River. The circular cladogram illustrates the fungal taxonomic hierarchy from phylum to genus level. Taxa with LDA score ≥ 2.0 and FDR-corrected *P* < 0.05 were identified as winter-enriched fungal biomarkers. Red, blue, and green branches correspond to the upstream CSY, midstream GZ, and downstream EL sites, respectively. Winter-indicator fungal taxa are mainly affiliated with Basidiomycota and Rozellomycota, with differential taxa widely distributed across the midstream GZ and downstream EL sites. Yellow nodes represent taxa without significant differential enrichment.
