# Supplementary material for: Responses and assembly mechanisms of planktonic bacterial and fungal communities in the upper Chishui River during dry and wet seasons
Source: Front Microbiol. 2026 Oct 20;17:1949002. doi: 10.3389/fmicb.2026.1949002 (PMC13727556; doi:10.3389/fmicb.2026.1949002)
Supplement: Supplementary file 1 [file Data_Sheet_1.pdf]

Supplementary Table S1

Table S1 PCR reaction system

Reagent	Volume
5× FastPfu Buffer	4μL
dNTPs	2μL
Forward primer (5 μmol·L^{-1})	0.8μL
Reverse primer (5 μmol·L^{-1})	0.8μL
FastPfu Polymerast	0.4μL
BSA	0.2μL
Template DNA	10ng
ddH_2O	Adjusted to 20 μL
Total volume	20μL
