# Supplementary material for: Responses and assembly mechanisms of planktonic bacterial and fungal communities in the upper Chishui River during dry and wet seasons
Source: Front Microbiol. 2026 Oct 20;17:1949002. doi: 10.3389/fmicb.2026.1949002 (PMC13727556; doi:10.3389/fmicb.2026.1949002)
Supplement: Supplementary file 2 [file Data_Sheet_2.pdf]

Table S2 **Variance inflation factor (VIF) values of environmental variables in wet and dry seasons, and variable retention decision for ordination analysis**

Variable	Wet-season VIF	Dry-season VIF	Retained (wet)	Retained (dry)
Water temperature	165.24256	52.97134		
pH	179.16235	40.43277	Yes	Yes
DO	139.31107	78.49997		
Conductivity	46.51019	64.62202		
TN	1383.86714	98.76366		
TP	130.11096	31.85667	Yes	Yes
NH_4^+–N	68.24286	39.37848	Yes	No (VIF > 10)
NO_3^-–N	927.95549	90.14449		
